# Exercise-responsive crosstalk within local muscle-bone units in osteosarcopenia: from mechanotransduction to secretome signalling

**DOI:** 10.3389/fphys.2026.1923562

**Published:** 2026-08-25

**Authors:** Zehao Guan, Xinyu Fu, Pai Zhang, Zongjian Luo, Hailong Wu, Qi Liu, Yitong Xu

**Affiliations:** 1 College of Traditional Chinese Medicine, Changchun University of Traditional Chinese Medicine, Changchun, Jilin, China; 2 Department of Orthopedics, Affiliated Hospital to Changchun University of Traditional Chinese Medicine, Changchun, Jilin, China; 3 Department of Andrology, First Affiliated Hospital of Tianjin University of Traditional Chinese Medicine, Tianjin, China

**Keywords:** endocrine factors, exercise training, local muscle-bone unit, mechanotransduction, osteosarcopenia

## Abstract

Osteosarcopenia is a geriatric syndrome characterized by the overlapping features of osteoporosis and sarcopenia. Its core pathology extends beyond parallel declines in bone and muscle mass. It also involves coordinated disturbances in muscle contractile capacity, skeletal mechanosensitivity, bone marrow cell-lineage commitment, and muscle- and bone-derived secretory signalling. Exercise represents a key non-pharmacological intervention that simultaneously targets skeletal muscle and bone. However, previous studies have mainly focused on changes in systemic bone mineral density, muscle mass, or circulating endocrine factors, whereas the site-specific adaptive relationship between particular muscle groups and adjacent skeletal sites has not been sufficiently integrated. This review aims to address the mechanisms underlying exercise-responsive crosstalk within local muscle–bone units in osteosarcopenia. We focus on the lumbar paraspinal muscle–vertebral body unit and the hip periarticular muscle–proximal femur unit, integrating evidence from imaging studies, animal experiments, exercise interventions, and endocrine mechanisms of muscle–bone communication. Current evidence suggests that exercise promotes adaptive remodeling of local muscle–bone units through site-specific mechanical loading and modulation of the local secretome. This includes both the paraspinal muscle–vertebral body unit and the hip periarticular muscle–proximal femur unit. The concept of exercise-responsive local muscle–bone units provides a translational framework for understanding site-specific degeneration in osteosarcopenia and for developing more precise exercise-based interventions.

## 1 Introduction

### 1.1 Osteosarcopenia

Osteoporosis(OP) and sarcopenia(SP) are common comorbid disorders in older adults and frequently coexist in clinical practice, substantially increasing the risk of falls, fractures, mortality, and accelerated ageing (Clynes et al., 2021; Teng et al., 2021). OP is a common musculoskeletal disease characterized by low bone mass and deterioration of bone microarchitecture, leading to reduced bone strength and increased fracture risk (Compston et al., 2019). SP is a progressive, generalized skeletal-muscle disorder defined by accelerated loss of muscle mass and function, and is associated with falls, functional decline, frailty, and mortality (Cruz-Jentoft et al., 2019). Both disorders typically occur in older populations. Loss of muscle strength occurs earlier and progresses more rapidly than many other age-related changes in body composition; reduced strength and impaired muscle quality further diminish the mechanical stimuli received by bone, adversely affecting bone mass, microarchitecture, and strength (Goodpaster et al., 2006).

The combined burden of OP and SP poses a substantial challenge to health-care systems and society. Current therapeutic research has mainly focused on pharmacological treatment, exercise, or their combination. Earlier reviews have often emphasised systemic inflammation, endocrine factors, or overall changes in bone mineral density(BMD) and muscle mass. Less attention has been given to an integrated framework that connects exercise stimuli, site-specific muscles, adjacent skeletal structures, and local molecular mechanisms. Exercise is an effective therapeutic strategy that can influence the progression of both SP and OP and reduce the economic burden on patients, families, and society. Yet the biological effects of exercise are not uniform: different exercise modalities recruit different muscle groups, and these muscle groups may have distinct effects on SP and OP. This review therefore focuses on local muscle-bone interactions under mechanical stimulation and the endocrine networks that regulate them.

### 1.2 Local muscle groups and local bone

The concept of the muscle-bone unit does not refer to the simple anatomical coexistence of muscle and bone. Rather, it emphasizes that, at a given anatomical site, bone mass and strength should be matched to the physiological loads generated by the corresponding muscles. Frost’s mechanostat theory proposes that bone continuously adjusts its mass and architecture according to local mechanical strain. Building on this theory, Schoenau developed the functional muscle-bone unit framework, arguing that under physiological conditions the largest mechanical loads acting on bone arise mainly from muscle contraction. This muscle contraction represents the dominant active (non-gravitational) mechanical load acting on bone. Therefore, bone mass or bone strength should not be interpreted independently of the muscle force or muscle cross-sectional area (CSA) at the corresponding site (Schoenau et al., 2002; Frost, 2003; Schönau, 2005). The relationship between site-matched bone mineral content (BMC) and muscle CSA can be used to characterize this functional coupling. The more relevant question is not simply whether bone mass is low, but whether the bone at a specific site is sufficient for the muscle force applied to that site, and whether that muscle force is appropriate for the individual’s body size (Schönau, 2005).

From the perspective of the muscle-bone unit, SP and OP are not two independent degenerative conditions that coincidentally coexist in older age. They are highly comorbid, share risk factors, and communicate through mechanical and secretory signalling. They may therefore represent coordinated impairment of the ‘load-generating’ and ‘load-bearing’ components within the same local functional system (Bonewald et al., 2013; Brotto and Bonewald, 2015; Reginster et al., 2016). Muscle degeneration manifests as reduced muscle mass, strength, and quality, resulting in weakened mechanical stimuli and abnormal myokine signalling. Bone degeneration manifests as impaired bone mass, microarchitecture, and strength, reducing the reserve capacity of bone to adapt to existing loads. Once the two conditions coexist, mechanical mismatch and biological crosstalk within the local muscle-bone unit(LMBU) may be amplified, creating a vicious cycle that increases skeletal fragility, functional decline, and the risk of falls and fractures.

Within the LMBU framework, mechanical loading and biochemical signalling should be viewed as complementary mechanisms. Contraction of anatomically matched muscles generates site-specific forces that shape the mechanical environment of adjacent bone. In parallel, muscle- and bone-derived secreted factors may regulate skeletal mechanosensitivity and the metabolic, inflammatory, and cell-fate context in which mechanical cues are interpreted. These pathways may also be interdependent: mechanical stimulation can modify the local secretome, whereas secreted factors may alter the capacity of muscle and bone to respond to subsequent loading. Mechanical loading may therefore confer anatomical specificity on muscle–bone coupling, while secretome signalling may modulate the magnitude and direction of the adaptive response. However, the relative contributions and temporal interactions of these mechanisms remain incompletely understood and may vary according to anatomical site, ageing, disease severity, and exercise modality.

In the pathological framework of SP complicated by OP, the muscle-bone unit is important not only because it explains their high comorbidity, but also because it indicates that muscle degeneration and bone fragility are mutually reinforcing processes along the same functional axis. Assessment should therefore move beyond isolated measures of muscle mass or BMD and should focus more on site-specific muscle-bone matching and its integrated clinical consequences.

## 2 The lumbar paraspinal muscle-vertebral body local muscle-bone unit

At the lumbar level, the muscle-bone unit can be specified as the lumbar paraspinal muscle-vertebral body LMBU. Its principal muscular components include the multifidus (MF), erector spinae (ES), and the psoas, which is frequently quantified alongside posterior paraspinal muscles. Its skeletal component comprises the adjacent vertebral bodies and their BMD or fragility phenotypes. The central issue is whether the biological indices of these components are appropriately matched. Clinically, the lumbar region, particularly the L3 vertebral level, has become the standard imaging landmark for the diagnosis of SP (Cruz-Jentoft et al., 2019). This anatomical level allows the simultaneous evaluation of muscle degeneration, vertebral bone quality, and local muscle–bone interactions. The lumbar paraspinal muscle–vertebral body LMBU represents a clinically relevant anatomical framework for investigating site-specific muscle–bone crosstalk in osteosarcopenia(OS).

### 2.1 Clinical imaging evidence

Current imaging evidence suggests that local muscle ‘quality’ may reflect local bone status more directly than muscle ‘quantity’ alone. Ma et al. applied the muscle-bone unit concept to the lumbar spine and found that both muscle mass and bone mass decreased with age; however, among individuals with low BMD, bone mass declined more markedly than muscle mass, meaning that relatively more muscle mass was present per unit of bone mass. This finding suggests that degenerated vertebrae may bear relatively higher local mechanical loads (Ma et al., 2014). Zhao et al. used chemical-shift water-fat magnetic resonance imaging (MRI) in 88 participants to measure proton density fat fraction (PDFF) of the ES, MF, and psoas at L2/3, L3/4, and L4/5, and measured L1-L3 BMD using quantitative computed tomography(QCT). Muscle PDFF was significantly lower in participants with normal BMD than in those with osteopenia or OP. After adjustment for age, sex, and body mass index (BMI), paraspinal muscle PDFF showed a moderate negative correlation with lumbar QCT-BMD, with standardized beta coefficients of approximately -0.21 to -0.29. The study suggests that paraspinal fatty infiltration may serve as an imaging biomarker of low lumbar BMD (Zhao et al., 2019).

In a QCT study of 367 participants, Li et al. measured mean L3-L5 BMD and quantified fat fractions of the psoas and ES at corresponding levels. Mean paraspinal muscle fat fraction correlated strongly and negatively with BMD (r = -0.721). Multivariable linear regression showed that vertebral fracture and lower BMD were independently associated with greater muscle fatty infiltration, indicating that the association between reduced muscle quality and vertebral bone loss cannot be explained by age alone (Li et al., 2022). Zhou et al. similarly used dual-energy computed tomography (DECT) and QCT and confirmed that fat content in the ES, MF, and psoas correlated negatively with L1-L2 volumetric BMD (vBMD). This study further suggested that the relationship between BMD and fat content may be stronger for the ES and MF than for the psoas (Zhou et al., 2022). Chiapparelli et al. subsequently reported, in 105 patients undergoing lumbar fusion, that MRI-derived paraspinal functional cross-sectional area(FCSA) at L3-L5 was associated with regional vBMD assessed by QCT at L1-L2 and the sacral ala. In women, L3 psoas FCSA was positively correlated with L1-L2 and S1 vBMD, while L3 posterior paraspinal muscle FCSA and L5 psoas FCSA were positively correlated with sacral ala vBMD; these associations were not observed in men. These findings indicate sex differences and regional specificity in the lumbar muscle-bone relationship (Chiapparelli et al., 2022).

Han et al. compared patients with degenerative lumbar spine disease who had normal BMD, osteopenia, or OP. MRI was used to assess fatty infiltration of the MF and ES and psoas FCSA, while bone status was assessed using dual-energy X-ray absorptiometry (DXA) and computed tomography (CT) Hounsfield units (HU). Patients with OP had greater MF and ES fatty infiltration than those with normal BMD, and these muscle-quality indices were negatively correlated with lumbar T-scores and mean lumbar HU values. The authors suggested that paraspinal degeneration should be considered when low bone mass is identified (Han et al., 2022). In 383 patients with lumbar disc herniation, Li et al. measured CSA and PDFF of the MF, ES, and psoas at L3/4, L4/5, and L5/S1, and measured L1 and L2 vBMD using QCT. The OP group had higher paraspinal PDFF and lower CSA; after adjustment for age and sex, MF PDFF and psoas CSA were independent factors associated with vBMD. This study supports the relevance of both muscle fatty degeneration and muscle CSA to lumbar bone status, while also showing that different muscle groups do not have identical imaging significance (Li et al., 2024). Within the lumbar LMBU, muscle-quality indices such as fatty infiltration may correspond more directly to vertebral fragility phenotypes than simple atrophy.

Higher-level integrative evidence is consistent with these findings. A 2023 meta-analysis including 11 studies showed that, compared with individuals without fractures, patients with incident osteoporotic vertebral fracture had smaller ES + MF CSA, smaller psoas CSA, and greater fatty infiltration. In recurrent vertebral fracture, increased fatty infiltration remained evident, whereas CSA differences were less consistent. The analysis suggested that paraspinal muscle degeneration may help identify individuals at high risk of vertebral fracture (Chen et al., 2023). Kim et al. retrospectively analyzed 262 patients with osteoporotic vertebral fracture and compared paraspinal muscle quality between single and multiple vertebral fractures. Using axial T2-weighted MRI at the L4 upper endplate, they measured muscle CSA and fatty degeneration. The multiple-fracture group had more severe fatty degeneration across all paraspinal muscles and lower lean muscle volume of the MF, psoas, and quadratus lumborum, suggesting that paraspinal muscle quality may contribute to vertebral fracture cascade progression (Kim et al., 2023). Xiong et al. included 307 patients with acute osteoporotic vertebral compression fracture, measured paraspinal CSA and fatty infiltration on L3/4 and L4/5 MRI, and developed a nomogram for predicting subsequent vertebral fracture. Percutaneous vertebroplasty (PVP), higher L3/4 psoas fatty infiltration, and lower L4/5 MF functional relative CSA were associated with subsequent fracture (Xiong et al., 2024). Tang et al. developed and validated a predictive model for new vertebral compression fractures after vertebral augmentation. Surgical method, spinal CT value, and MF skeletal muscle index were independent predictors of new compression fractures after PVP or percutaneous kyphoplasty (PKP), with areas under the curve (AUCs) of 0.801, 0.664, and 0.832 in the training, validation, and external validation cohorts, respectively (Tang et al., 2024).

It should be noted that not all paraspinal imaging indices predict fracture independently of BMD. A 2024 dual-layer detector CT study showed that the effective atomic number of paraspinal muscles was associated with lower acute vertebral-fracture risk in older men, but muscle density, area, and effective atomic number were no longer independent risk factors after further adjustment for vBMD (Mai et al., 2024). Thus, paraspinal imaging measures currently have value as auxiliary risk-stratification tools rather than as substitutes for BMD or as established independent diagnostic criteria.

Overall, imaging studies support a clinically meaningful association between local paraspinal muscle degeneration and vertebral bone loss, but they do not establish causality. MRI-, QCT-, and DECT-based studies consistently show that higher fatty infiltration or PDFF of the MF, ES, and psoas is associated with lower lumbar BMD or vBMD, and that reduced FCSA is associated with poorer regional bone quality. Importantly, this relationship is both muscle-specific and site-specific. The MF, as a deep segmental stabilizer, may reflect local spinal stability and vertebral fragility more closely. The ES represents the posterior extensor system and is often analyzed together with the MF. The psoas, although widely used as an opportunistic marker of SP, may reflect systemic muscle mass and lumbopelvic function more than pure posterior extensor capacity. Paraspinal imaging parameters, particularly fatty infiltration and FCSA, show potential for stratifying the risk of incident osteoporotic vertebral fracture, recurrent vertebral fracture, multiple vertebral fractures, and new compression fractures after vertebral augmentation. However, because most studies are cross-sectional or retrospective and because some muscle indices lose independent predictive value after adjustment for vBMD, paraspinal imaging should currently be presented as complementary to bone-density and bone-quality assessment, rather than as a replacement for traditional skeletal-strength measures.

### 2.2 Exercise intervention

Human intervention studies rarely include lumbar paraspinal MRI measures and adjacent vertebral vBMD as co-primary endpoints, but their direction is broadly consistent with the mechanistic evidence. In a 1993 RCT, postmenopausal women were randomized to psoas training or deltoid-training control, and lumbar cancellous BMD was assessed by CT. In the overall analysis, bone loss was lower in the training group but did not reach statistical significance. In the high-compliance subgroup, however, mean cancellous BMD across four lumbar vertebrae declined significantly in controls over 12 months, whereas it was largely maintained in the psoas-training group, producing a significant between-group difference. The study supports the hypothesis that physical activity may have a local effect on vertebral bone and suggests that traction or contraction loading from muscles adjacent to the lumbar spine may provide local skeletal protection (Revel et al., 1993). Sinaki et al. followed postmenopausal women for 10 years after a two-year randomized back-extensor strengthening program. The training group maintained greater back-extensor strength at 10 years, showed statistically significant between-group differences in BMD, and had a lower incidence of vertebral compression fracture (1.6% versus 4.3%; control relative risk approximately 2.7) (Sinaki et al., 2002).

Subsequent RCTs have consistently shown that back-extensor or trunk-muscle training can improve local functional outcomes in women with OP. Hongo et al. showed that four months of low-intensity back exercise improved back-extensor strength and quality of life in 80 postmenopausal women with OP (Hongo et al., 2007). Cergel et al. showed, in 60 women with osteoporotic vertebral fracture, that six weeks of supervised back-extensor strength training improved pain, strength, endurance, functional mobility, and quality of life more than home exercise or control (Çergel et al., 2019). In a higher-quality RCT of 101 postmenopausal women with low bone mass, an eight-month high-intensity resistance and impact training (HiRIT) program (twice weekly, 30 min/session, 5 sets of 5 repetitions) increased lumbar BMD by 2.9%, whereas controls declined by 1.2%. Femoral neck (FN) BMD, FN cortical thickness, height, and all functional performance measures also improved more than in controls. For the lumbar LMBU, this supports the view that high-magnitude and high-rate loading is more likely than low-intensity activity to trigger skeletal adaptation, although it does not prove that the effect is mediated by morphological improvement of a specific paraspinal muscle (Watson et al., 2018).

In older community-dwelling men aged over 72 years with SP and osteopenia or OP, a 54-week supervised high-intensity dynamic resistance-training program performed twice weekly, with both groups receiving protein, vitamin D, and calcium supplementation, reduced lumbar QCT-BMD loss. QCT-BMD decreased by 2.5% in controls but increased by 1.6% in the exercise group, with a significant between-group difference; skeletal muscle mass index also improved (Kemmler et al., 2020). A subsequent Dixon MRI analysis quantified fatty infiltration, muscle tissue volume, and intermuscular adipose tissue (IMAT) of the psoas and ES. Sixteen months of high-intensity resistance training did not improve psoas or ES fatty infiltration. The authors suggested that, despite lumbar BMD benefit in the same cohort, paraspinal fatty infiltration may be less exercise-responsive or difficult to reverse in older men with OS. Thus, lumbar bone can respond to high-intensity training, but local paraspinal fatty infiltration does not necessarily improve synchronously. This study suggests that bone tissue and local paraspinal muscles may exhibit different temporal dynamics in their response to exercise stimulation; that is, whilst lumbar BMD may improve, the fatty infiltration of local paraspinal muscles may not necessarily be reversed at the same rate (Kircher et al., 2024).

Higher-level evidence also supports lumbar skeletal benefit. The 2023 systematic review and meta-analysis by Mohebbi et al. included 80 exercise-intervention studies involving 5,581 postmenopausal women and showed significant exercise-induced improvements in lumbar spine, FN, and total hip (TH) BMD, with standardized mean differences (SMDs) of 0.29, 0.27, and 0.41, respectively (Mohebbi et al., 2023). This supports the clinical conclusion that the lumbar spine and proximal femur are responsive to exercise-induced mechanical stimuli. However, this meta-analysis was based mainly on DXA-derived areal BMD (aBMD) and did not systematically assess paraspinal morphology, local muscle quality, vertebral microstructure, or muscle-bone molecular crosstalk. It is therefore best interpreted as evidence for the clinical efficacy of exercise on BMD, not as proof of a local muscle-mediated causal pathway. Heterogeneity in lumbar BMD outcomes also suggests that exercise prescription in OS should emphasize training intensity, progressive loading, professional supervision, and anatomical site specificity rather than a general increase in physical activity.

Human exercise evidence therefore supports the lumbar paraspinal-vertebral LMBU as a clinically meaningful but not yet definitive framework. The most anatomically specific evidence comes from the psoas-training RCT in postmenopausal women, where high adherence preserved CT-measured lumbar cancellous BMD better than control. In contrast, early low-load, non-weight-bearing back-extensor training improved back-extensor strength but did not clearly attenuate lumbar bone loss over two years, indicating that strength gains alone do not necessarily translate into measurable BMD benefit and that loading may need to reach an osteogenic mechanical threshold. The 10-year follow-up of that cohort, however, showed sustained extensor strength, more favorable lumbar BMD, and fewer vertebral compression fractures. This observation suggests that tissue remodeling exhibits temporal differences. Muscle and bone may respond to exercise over different timescales and through distinct adaptive processes. Mechanical loading generated by muscle contraction is transmitted to adjacent bone and regulates skeletal remodeling, whereas muscle- and bone-derived biochemical signals may further modulate these adaptive responses. Unchanged paraspinal muscle fatty infiltration despite improved lumbar BMD may therefore reflect differences in tissue-specific remodeling dynamics and the sensitivity of imaging endpoints, rather than true dissociation between muscle and bone adaptation. Further longitudinal studies are required to clarify the temporal relationship between muscular and skeletal adaptation to exercise. More recent high-intensity, multi-component, or dynamic resistance programs, including LIFTMOR, and FrOST, show that supervised resistance, impact, or dynamic loading can improve trunk extensor strength, physical function, and lumbar BMD, including QCT-measured vertebral BMD in older adults with OS. Yet the FrOST MRI analysis found no stable improvement in ES or psoas fatty infiltration despite lumbar BMD gain, implying possible dissociation between vertebral osteogenic adaptation and paraspinal compositional change in very old patients with OS.

### 2.3 Preclinical mechanistic evidence

Mechanistic studies provide stronger support for the idea that local muscle groups can directly shape adjacent bone status. Local paraspinal disuse or atrophy can deteriorate adjacent lumbar bone, while exercise training can improve lumbar BMD, mechanical properties, and osteogenic or adipogenic signals in ovariectomized (OVX) or disuse models. These effects are influenced by age, exercise intensity, nutritional status, and baseline musculoskeletal function. Aliprantis et al. induced unilateral muscle paralysis in mice using botulinum toxin and found marked ipsilateral trabecular bone loss within days. Local receptor activator of nuclear factor kappa-B ligand (RANKL) increased by 113% at day 7, osteoclast numbers increased by 122% at day 5 compared with the contralateral side, and human recombinant osteoprotegerin (hrOPG) or conditional nuclear factor of activated T cells c1 (NFATc1) deletion significantly blocked this bone loss (Aliprantis et al., 2012). Poliachik et al. further showed that applying pulsed focused ultrasound to paralyzed muscle, without acting directly on bone, attenuated adjacent bone loss (Poliachik et al., 2014). Although these two classic mechanistic experiments used limb models, later studies in Sprague-Dawley (SD) rats showed that local botulinum toxin-A injection into paravertebral muscles also reduced adjacent lumbar BMD and mechanical properties, providing more direct site-specific evidence for the lumbar LMBU (Wang et al., 2018).

Animal exercise studies also support a lumbar response to mechanical stimulation. In three-month-old female Wistar rats allocated to sham control, sedentary OVX, or exercised OVX groups, treadmill training for 12 weeks (4 days/week, 1 h/day; 15 m/min for the first 6 weeks and 19 m/min for the final 6 weeks) was followed by morphological and compression testing of L4. OVX reduced vertebral height and maximum breaking load, whereas treadmill exercise increased vertebral mass and improved stiffness compared with sedentary OVX rats. This study provides basic evidence that sustained dynamic exercise can improve lumbar mechanical performance in estrogen-deficiency-induced OP (Simões et al., 2008). In another study, three-month-old female SD rats were assigned to sham, OVX, OVX exercise, or OVX estrogen-replacement groups. DXA, histology, immunohistochemistry, and western blotting were used to assess BMD, peroxisome proliferator-activated receptor gamma(PPARγ) and Runx2 expression, and intraosseous lipid deposition in tibia and lumbar spine. OVX reduced lumbar and femoral BMD, increased marrow fat vacuoles, upregulated PPARγ, and increased bone triglyceride content. Treadmill exercise reversed these changes but did not significantly enhance Runx2 expression, suggesting that the lumbar bone-protective effect of exercise may act less through direct upregulation of Runx2 and more through inhibition of PPARγ-mediated marrow adipogenesis and rebalancing of bone marrow mesenchymal stem/stromal cell(BMMSC) osteogenic versus adipogenic differentiation (Chen et al., 2011).

Building on this model, a related study analyzed glycogen synthase kinase-3 beta (GSK-3β)/β-catenin signalling, PPARγ, osterix, and vertebral ultimate strength in sham, OVX, OVX exercise, and estrogen-replacement groups. Compared with sedentary OVX rats, treadmill-trained rats had higher lumbar ultimate strength, increased β-catenin and phosphorylated GSK-3β expression, and lower PPARγ expression. The effect was not explained by systemic estrogen-like action, indicating that treadmill exercise can locally activate GSK-3β/β-catenin signalling and suppress adipogenic signalling in lumbar vertebrae, thereby improving mechanical strength (Bu et al., 2012). In another OVX model, moderate-intensity treadmill training for 17 weeks (main phase: 20 m/min, 60 min/day, 5 days/week) improved body composition, BMD, and bone microarchitecture. In the reported data, trunk muscle percentage fell after OVX and partially recovered with exercise, while L4 BMD increased from approximately 0.199 g/cm2 in OVX rats to 0.223 g/cm2 in exercised rats. Transcriptomic analysis of peripheral blood mononuclear cells suggested that the phosphoinositide 3-kinase (PI3K)-Akt pathway and factors such as CCL2, Nos3, and Tgfb3 may participate in exercise-related bone protection (Gao et al., 2021).

The lumbar skeletal response to exercise appears context-dependent. In 15-week-old female Wistar rats, OVX reduced lumbar and femoral BMD and increased bone turnover. Exercise plus calcium supplementation had the strongest protective effect on L2-L4 and femoral BMD, whereas exercise alone was more effective at the younger or shorter intervention stage and less effective with longer duration or ageing. This supports the more cautious interpretation that lumbar benefit requires an adequate intervention window and may depend on nutritional and systemic metabolic background (Gala et al., 2001). In 24-month-old male SD rats, 8 weeks of treadmill training suppressed bone-marrow NOD-like receptor protein 3(NLRP3)/caspase-1/interleukin-1 beta(IL-1β) inflammatory signalling and improved lower-limb BMD and microarchitecture, but did not significantly improve L5 BMD or microarchitecture compared with aged controls. The authors suggested that insufficient duration or speed, ageing, reduced skeletal responsiveness in a sarcopenic background, and limited sample size may explain the negative lumbar findings. This study indicates that the lumbar spine may have a higher threshold for exercise-induced skeletal response, particularly under ageing and SP conditions (Wu et al., 2022). In an OVX plus tail-suspension model simulating bone loss and muscle disuse, treadmill exercise (12m/min, 60 min/day, 5 days/week) improved femoral BMD, femoral metaphysis trabecular bone strength, cortical bone structure and strength at the femoral diaphysis. In addition, the intervention increased the percentage of whole-body skeletal muscle mass while reducing whole-body fat mass and bone marrow adipose tissue. Teriparatide plus exercise had stronger skeletal and mechanical effects. Because this model did not pair MF, ES, or psoas changes with adjacent lumbar bone measures, it should be used as supportive evidence for coordinated bone-muscle adaptation rather than as direct evidence for a paraspinal-vertebral mechanism (Sato et al., 2021). Whole-body vibration at 90 Hz for 35 days in OVX rats improved vertebral mechanical performance and trabecular structure, suggesting that vertebral trabecular bone is responsive to external mechanical stimulation, although this differs from active exercise (Sehmisch et al., 2009).

Taken together, animal studies provide an important mechanistic bridge between imaging associations and the biological plausibility of a lumbar paraspinal-vertebral LMBU. Direct exercise studies that simultaneously quantify adaptation of the MF, ES, or psoas and adjacent vertebral microstructure remain limited. Nevertheless, several models support a coherent causal framework: local paraspinal dysfunction induced by botulinum toxin injection leads to reduced lumbar BMD, impaired trabecular architecture, and lower compression strength, showing that loss of regional muscle loading is sufficient to deteriorate adjacent vertebrae. Conversely, treadmill exercise in OVX rats improves lumbar bone mass, stiffness, ultimate strength, and BMD, accompanied by inhibition of PPARγ-mediated adipogenic signalling and activation of GSK-3β/β-catenin osteogenic signalling. Moderate treadmill training can also partially restore trunk muscle proportion and L4 BMD, suggesting coordinated trunk musculoskeletal adaptation, although this does not prove coupling at the level of an individual paraspinal muscle. Existing animal evidence therefore supports the lumbar paraspinal-vertebral unit as an exercise-sensitive but threshold-dependent local system in which muscle-derived mechanical loading and marrow-lineage regulation jointly influence vertebral quality.

### 2.4 Summary

In summary, human evidence supports the lumbar paraspinal-vertebral unit as an exercise-responsive local functional system, but it is insufficient to prove that imaging improvement of the MF, ES, or psoas directly enhances adjacent vertebral strength. Future trials should integrate targeted paraspinal training, quantitative muscle imaging, QCT or HR-pQCT vertebral assessment, biomechanical modelling, and fracture outcomes within the same longitudinal design. At present, the lumbar LMBU is best understood as a site-specific functional framework in which local muscle quality, mechanical load transfer, and vertebral adaptation are closely related, while muscle-specific causal pathways remain to be verified (**Table 1**).

**Table 1:** Evidence for the lumbar paraspinal muscle-vertebral body local muscle-bone unit.

A. Lumbar imaging and fracture-risk evidence														
Author Year			Study type	Population/model			Muscle-bone assessment target		Methods			Main findings		No.
Ma et al. 2014			Cross-sectional imaging study	Adult participants with varying BMD			Lumbar muscle mass and lumbar bone mass		Assessment of lumbar BMC and muscle CSA/muscle mass			Both bone mass and muscle mass decreased with age; in individuals with low BMD, bone mass declined more markedly than muscle mass, suggesting local muscle-bone mismatch.		(Ma et al., 2014)
Zhao et al. 2019			Cross-sectional study	88 participants			ES, MF, psoas and lumbar BMD		Chemical-shift MRI to measure PDFF at L2/3, L3/4 and L4/5; QCT to measure L1-L3 BMD			PDFF of the three muscle groups showed a moderate negative correlation with QCT-BMD, suggesting that paraspinal fatty infiltration may serve as an imaging marker of low BMD.		(Zhao et al., 2019)
Li X et al. 2022			Cross-sectional study	367 participants			Paraspinal muscle fat fraction and L3-L5 BMD		QCT-based BMD measurement and quantification of psoas and ES fat fraction			Mean paraspinal muscle fat fraction was strongly and negatively correlated with BMD; vertebral fracture and low BMD were independent correlates of increased fatty infiltration.		(Li et al., 2022)
Zhou et al. 2022			Cross-sectional DECT/QCT study	–			Fat content of ES, MF and psoas, and L1-L2 vBMD		Fast kVp-switching DECT combined with QCT			Fat content in all three muscle groups was negatively correlated with vBMD; correlations were stronger for ES and MF than for psoas.		(Zhou et al., 2022)
Chiapparelli et al. 2022			Cross-sectional study	105 patients undergoing lumbar fusion			Paraspinal and psoas FCSA, and regional vBMD		MRI assessment of L3-L5 FCSA; QCT assessment of L1-L2 and sacral vBMD			In the female subgroup, FCSA was positively correlated with regional vBMD, whereas no significant association was observed in men, indicating sex- and region-specific relationships.		(Chiapparelli et al., 2022)
Han et al. 2022			Case-control study	Patients with degenerative lumbar spine disease (normal BMD, osteopenia or osteoporosis)			MF/ES fatty infiltration, psoas FCSA, DXA T-score and lumbar CT HU values		MRI, DXA and CT HU assessment			Patients with osteoporosis had greater MF and ES fatty infiltration, which was negatively correlated with T-scores and mean HU values.		(Han et al., 2022)
Li Z et al. 2024			Cross-sectional study	383 patients with lumbar disc herniation			CSA and PDFF of MF, ES and psoas; L1-L2 vBMD		MRI assessment of CSA/PDFF at L3/4-L5/S1; QCT assessment of vBMD			The osteoporosis group had higher PDFF and lower CSA; after adjustment for age and sex, MF PDFF and psoas CSA were independent factors associated with vBMD.		(Li et al., 2024)
Chen et al. 2023			Meta-analysis	11 studies			Paraspinal muscle imaging parameters and incident/recurrent osteoporotic vertebral fracture		Meta-analysis			Compared with individuals without fracture, patients with OVF had smaller ES-MF and psoas CSA and higher fatty infiltration; increased fatty infiltration was more evident in recurrent fracture.		(Chen et al., 2023)
Kim et al. 2023			Retrospective study	262 patients with osteoporotic vertebral fracture			Paraspinal muscle quality and single/multiple vertebral fractures		T2-weighted MRI at the L4 upper endplate to measure CSA and fatty degeneration			The multiple-fracture group had more severe paraspinal fatty degeneration and lower lean muscle volume of MF, psoas and quadratus lumborum.		(Kim et al., 2023)
Xiong et al. 2024			Retrospective prediction study	307 patients with acute osteoporotic vertebral compression fracture			Psoas/MF features and subsequent vertebral fracture		MRI assessment at L3/4 and L4/5; nomogram construction			PVP treatment, higher L3/4 psoas fatty infiltration and lower L4/5 MF functional relative CSA were associated with subsequent fracture.		(Xiong et al., 2024)
Tang et al. 2024			Retrospective model development and validation study	Patients after PVP/PKP			MF parameters and new postoperative vertebral compression fracture		Model development using spinal CT values and paraspinal muscle parameters			Surgical method, spinal CT value and MF skeletal muscle index were independent predictors; AUCs were 0.80, 0.66 and 0.832 in the training, validation and external validation cohorts, respectively.		(Tang et al., 2024)
Mai et al. 2024			Cross-sectional case-control study	Older patients assessed for acute vertebral fracture risk			Paraspinal muscle effective atomic number, density and area, and fracture risk		Dual-layer detector CT with adjustment for vBMD			In older men, a higher effective atomic number was associated with lower acute vertebral fracture risk; after adjustment for vBMD, muscle indices no longer independently predicted risk.		(Mai et al., 2024)

B. Human exercise-intervention and integrative evidence														
Author	Study type				Population/model			Muscle-bone assessment target			Methods		Main findings	No.
Revel et al. 1993	Randomised controlled trial				Postmenopausal women			Psoas-lumbar cancellous bone			Psoas training versus deltoid-training control; CT measurement of lumbar cancellous BMD		Overall differences were limited, but in the high-compliance subgroup, the training group showed better preservation of lumbar BMD after one year, supporting a local bone-protective effect of muscle-derived loading.	(Revel et al., 1993)
Sinaki et al. 2002	Prospective 10-year follow-up				Postmenopausal women			Back-extensor strength, lumbar BMD and vertebral fracture			Ten-year follow-up after completion of a two-year randomised back-extensor strengthening programme		The training group maintained greater back-extensor strength, had more favourable BMD, and showed a lower incidence of vertebral compression fractures.	(Sinaki et al., 2002)
Hongo et al. 2007	Randomised controlled trial				80 postmenopausal women with osteoporosis			Back-extensor function and quality of life			Four-month low-intensity back-exercise programme		The intervention improved back-extensor strength and quality of life.	(Hongo et al., 2007)
Cergel et al. 2019	Randomised controlled study				Postmenopausal women with osteoporosis			Back-extensor training-related outcomes			Six-week supervised training versus home exercise versus control		The supervised training group showed greater improvements in pain, strength, endurance, functional mobility and quality of life.	(Çergel et al., 2019)
Watson et al. 2018	Randomised controlled trial				101 postmenopausal women with low bone mass			Lumbar spine/femoral neck BMD and function			Eight-month high-intensity resistance and impact training (twice weekly, 5 sets x 5 repetitions)		Lumbar BMD increased by 2.9% in the intervention group versus a 1.2% decrease in controls; femoral neck BMD, cortical thickness and functional outcomes were also superior.	(Watson et al., 2018)
Kemmler et al. 2020	Randomised controlled trial				Community-dwelling older men (>=72 years) with osteosarcopenia			Lumbar QCT-BMD and SMI			Fifty-four-week supervised dynamic high-intensity resistance training with concurrent protein, vitamin D and calcium supplementation		Lumbar QCT-BMD increased by 1.6% in the exercise group and decreased by 2.5% in controls; SMI was also more favourable in the exercise group.	(Kemmler et al., 2020)
Kircher et al. 2024	Longitudinal MRI analysis nested within an RCT				Older men from the FrOST cohort			Psoas/ES fatty infiltration, muscle volume and IMAT			Sixteen-month follow-up using Dixon MRI		Despite previously reported lumbar BMD differences in the FrOST cohort, no clear improvement in psoas or ES fatty infiltration was observed, suggesting that vertebral bone adaptation and paraspinal compositional changes may not occur synchronously.	(Kircher et al., 2024)
Mohebbi et al. 2023	Systematic review and meta-analysis				80 studies including 5,581 postmenopausal women			Lumbar spine, femoral neck and hip BMD			Updated systematic review and meta-analysis		Exercise benefits involved lumbar spine, femoral neck and hip BMD, supporting an overall beneficial effect in postmenopausal osteoporosis, but not directly proving local muscle-specific prevention or treatment effects.	(Mohebbi et al., 2023)

C. Animal experiments and local mechanistic evidence														
Author		Study type		Population/model		Muscle-bone assessment target				Methods		Main findings		No.
Aliprantis et al. 2012		Animal mechanistic experiment		Mice		Local muscle paralysis and adjacent bone loss				Single-dose toxin-induced muscle atrophy; hrOPG intervention/NFATc1 conditional deletion; histological analysis		Adjacent bone loss occurred rapidly after muscle paralysis, with increased RANKL and osteoclasts; OPG or NFATc1 deficiency blocked adjacent bone loss.		(Aliprantis et al., 2012)
Poliachik et al. 2014		Animal experiment		Mouse paralysis model		Paralysed muscle-adjacent bone				Pulsed focused ultrasound treatment applied to paralysed muscle, with adjacent bone assessment		Even without direct action on bone, mechanical stimulation of paralysed muscle attenuated adjacent bone loss.		(Poliachik et al., 2014)
Wang X et al. 2018		Animal experiment		SD rats		Paraspinal muscle dysfunction and lumbar vertebral bone quality				Chronic focal botulinum toxin-A injection; assessment of BMD, trabecular bone and bone content		Local botulinum toxin-A injection into paraspinal muscles reduced adjacent lumbar BMD, bone quality and mechanical properties.		(Wang et al., 2018)
Simoes et al. 2008		Animal exercise intervention		Three-month-old female Wistar OVX rats		Lumbar vertebral bodies				Twelve-week treadmill training; L4 morphometric and compression testing		Exercise increased vertebral mass and stiffness and improved vertebral mechanical properties.		(Simões et al., 2008)
Chen Y et al. 2011		Animal exercise intervention		Three-month-old female SD OVX rats		Lumbar spine/femur and marrow adipogenesis				DXA, histology, immunohistochemistry and western blotting for PPARγ/Runx2		Treadmill exercise prevented bone loss and inhibited PPARγ expression and marrow adipogenesis, but did not significantly enhance Runx2 expression.		(Chen et al., 2011)
Bu et al. 2012		Animal exercise intervention		Three-month-old female SD OVX rats		Lumbar vertebral bodies				Treadmill training; assessment of GSK-3β/β-catenin, PPARγ, osterix and mechanical strength		Exercise increased lumbar ultimate strength, increased GSK-3β and phosphorylated β-catenin expression, and downregulated PPARγ.		(Bu et al., 2012)
Gao et al. 2021		Animal exercise intervention		Three-month-old female SD OVX rats		Trunk muscle proportion and L4 BMD				Seventeen-week moderate-intensity treadmill training; DXA, micro-CT and PBMC transcriptomics		Exercise improved body composition, L4 BMD and bone microarchitecture; PI3K-Akt and CCL2/Nos3/Tgfb-related pathways may be involved.		(Gao et al., 2021)
Gala et al. 2001		Animal exercise-intervention study		Fifteen-week-old female Wistar OVX rats		Lumbar L2-L4 and femoral BMD				Exercise, calcium supplementation and combined intervention; BMD and bone-turnover markers assessed at 13 and 20 weeks		The protective effect of exercise combined with calcium supplementation was stronger than exercise alone; exercise effects were influenced by age and intervention duration.		(Gala et al., 2001)
Wu et al. 2022		Animal exercise intervention		Twenty-four-month-old male SD aged rats		Bone-marrow inflammatory signalling, lower-limb bone and lumbar spine				Eight-week treadmill training; assessment of serum, bone marrow, femur, tibia and lumbar vertebrae		Exercise inhibited NLRP3/caspase-1/IL-1β inflammatory signalling and improved lower-limb bone, whereas L5 vertebral improvement was not significant.		(Wu et al., 2022)
Sato et al. 2021		Animal combined-intervention study		OVX and tail-suspended rats		FemoralBMD, skeletal muscle and fat				Eight-week treadmill exercise, teriparatide or combined intervention; assessment of BMD, bone strength and muscle strength		Exercise improved BMD, bone strength and muscle proportion and reduced fat mass; combined treatment with teriparatide produced greater effects.		(Sato et al., 2021)
Sehmisch et al. 2009		Animal mechanistic testing study		OVX osteopenic rats		Lumbar vertebral bodies				Low-magnitude high-frequency mechanical stimulation at 90 Hz for 35 days; biomechanical, morphometric and BMD assessment		Mechanical stimulation improved vertebral mechanical properties and trabecular structure, with some indices approaching those of the sham-operated group.		(Sehmisch et al., 2009)

PDFF, proton density fat fraction; CSA, cross-sectional area; FCSA, functional cross-sectional area; BMD, bone mineral density; BMC, bone mineral content; vBMD, volumetric bone mineral density; QCT, quantitative computed tomography; DXA, dual-energy X-ray absorptiometry; DECT, dual-energy computed tomography; MRI, magnetic resonance imaging; CT, computed tomography; HU, Hounsfield units; ES, erector spinae; MF, multifidus; OVF, osteoporotic vertebral fracture; PVP, percutaneous vertebroplasty; PKP, percutaneous kyphoplasty; SD, Sprague–Dawley; OVX, ovariectomised; PBMC, peripheral blood mononuclear cell; FN, femoral neck; IMAT, intermuscular adipose tissue; SMI, skeletal muscle mass index; FrOST, Franconian Osteopenia and Sarcopenia Trial; AUC, area under the curve.

## 3 The peri-hip muscle-proximal femur local muscle-bone unit

The hip differs from many skeletal sites where only BMD change can be observed. The proximal femur has clearly defined local muscle attachments, including the gluteus medius/minimus, gluteus maximus, iliopsoas, and short external rotators; mature DXA, QCT, and CT quantification methods; and abundant hard-outcome evidence, including first hip fracture, contralateral second hip fracture, and postoperative mortality. Hip evidence is therefore anatomically specific, quantitatively mature, clinically outcome-based, and relatively rich in exercise-loading studies. It can address not only whether local muscle is associated with local bone mass, but also whether this coupling affects fracture occurrence, fracture type, and prognosis.

### 3.1 Clinical evidence

Cross-sectional studies already show clear site specificity in this coupling. In 321 participants from the Tasmanian Older Adult Cohort, Ahedi et al. found that the muscles associated with hip and FN BMD were not all peri-hip muscles, but mainly the hip flexors (pectineus, sartorius, and iliopsoas) and some short external rotators (obturator externus and quadratus femoris). In contrast, the gluteus maximus and piriformis were not significantly associated with BMD. These findings suggest anatomical specificity in proximal femoral responses to muscle input; local bone does not uniformly ‘read’ signals from all neighboring muscles (Ahedi et al., 2014). Pasco et al. further showed, in 863 women, that hip flexor strength and hip abductor strength were positively associated with TH BMD, but that these associations disappeared after adjustment for total or appendicular lean mass. This indicates that the relationship between local hip strength and hip bone status is partly embedded within a broader body-composition framework (Pasco et al., 2015). Nevertheless, Capato et al. observed in 94 independent women over 60 years old that hip abductor peak torque was independently associated with FN BMD and multiple functional tests, indicating that gluteus medius or abductor function retains local bone-muscle relevance in older women (Capato et al., 2023). Together, these studies show that hip muscle-bone coupling exists, but is not simply a parallel decline in muscle mass and bone mass. It appears instead to represent functional pairing between selected muscle groups and proximal femoral loading pathways.

When the endpoint shifts from BMD correlation to actual fracture, local muscle quality becomes more important than muscle quantity alone. In a CT study of 438 patients with low-energy acute hip fracture and 316 healthy controls, Wang et al. found that gluteal muscle area, gluteal muscle density, and bone parameters were lower in the fracture group. For discriminating first hip fracture, gluteus medius/minimus density outperformed FN and TH aBMD, with AUCs of 0.88 for FN fracture and 0.95 for intertrochanteric fracture. Crucially, model performance fell significantly when muscle density was removed, suggesting that peri-hip muscle density provides fragility information not captured by BMD (Wang et al., 2020a). This concept is supported by fracture-subtype studies. In 554 older women, Huang et al. compared FN fracture (FNF) with trochanteric fracture (TRF) and found that overall muscle parameters were higher in the FNF group than in the TRF group. After adjustment for age, BMI, and TH aBMD, several gluteal parameters remained significantly associated with TRF, particularly among those younger than 80 years. Local gluteal degeneration may therefore contribute to fracture stratification beyond BMD, especially for intertrochanteric phenotypes that depend more strongly on the mechanical environment of the trochanteric cortex and tendon-attachment region (Huang et al., 2024).

More important than the mere association between gluteal fatty infiltration and fracture is the observation that, at the hip, local muscle quality may be closer to the true fracture phenotype than conventional aBMD. Ma et al. used CT to assess peri-hip muscle radiodensity and analyzed its relationship with hip fracture type and severity. Radiodensity of the gluteus maximus, gluteus medius/minimus, and adductor group differed across fracture types, whereas CSA did not. Higher muscle radiodensity, reflecting less fatty infiltration, was more often associated with undisplaced FNF, whereas lower radiodensity was associated with displaced FNF or intertrochanteric fracture. This study reinforces the concept that muscle-quality indices outperform simple muscle area, although its retrospective design and lack of routine BMD data mean that it should be interpreted as supportive rather than definitive evidence (Ma et al., 2026).

Prospective follow-up studies add a time dimension to this relationship. In a 2022 prospective cohort, Wang et al. showed that after first hip fracture, contralateral second-fracture risk was independently associated not only with aBMD but also with local gluteal muscle density. Gluteus medius/minimus density showed the strongest association (hazard ratio [HR] 1.68) and remained significant after additional adjustment for FN or TH aBMD (Wang et al., 2022). In 2024, the same group further reported that prediction of second fracture is time-dependent: within the first year, FN aBMD was the stronger predictor (HR 5.88), whereas from the second year onwards, gluteus maximus and gluteus medius/minimus density better predicted contralateral fracture (HR approximately 2.1) (Wang et al., 2024). These findings indicate that bone and muscle contributions within the same local axis are not fixed. Early risk appears to reflect immediate fragility driven by insufficient skeletal reserve, whereas later risk may reflect muscle fatty infiltration, impaired load-transfer efficiency, and weakened fall-protection responses.

A similar dual-channel pattern is seen for mortality. In 459 patients with hip fracture, Wang et al. reported that low gluteus maximus area, low gluteus maximus density, and low gluteus medius/minimus density were associated with poorer survival. One-year mortality was associated with gluteus maximus density (adjusted HR 1.83) and gluteus medius/minimus density (adjusted HR 1.98), whereas mortality after two years and beyond was more closely associated with gluteus maximus area (Wang et al., 2023). Ge et al. showed that bone-side information was also independently prognostic: in 394 older patients with hip fracture, higher trochanteric aBMD was associated with lower mortality risk, with an adjusted HR of 0.21 (Ge et al., 2023). From a prognostic perspective, the hip LMBU is therefore not an either-or model of muscle versus bone. It is better conceptualized as a dual-channel risk system formed by skeletal reserve and muscle quality.

### 3.2 Animal evidence

Animal studies further support local loading effects on the proximal femur, although they rarely measure individual peri-hip muscles. Peng et al. used an OVX rat model and found that OVX reduced the maximum breaking load of the FN, while exercise almost completely prevented the loss of mechanical strength in the FN, tibia, and humerus and partially overcame trabecular bone loss. Although gluteus medius or iliopsoas was not specifically measured, the study directly shows that exercise loading can protect FN strength under estrogen deficiency (Peng et al., 1994). Libouban et al. injected botulinum toxin into the quadriceps of mice to induce unilateral lower-limb paralysis and used micro-CT for long-term quantitative assessment of muscle and bone wasting. Paralyzed-side muscle atrophy and ipsilateral trabecular bone loss changed in a time-related manner, with partial late recovery. Although the skeletal sites were mainly the distal femur and tibia rather than the proximal femur, the study indicates that local muscle disuse is sufficient to drive ipsilateral bone loss (Libouban et al., 2018). Tang et al. used weight-bearing treadmill training in OVX rats and showed improved femoral BMD, trabecular microarchitecture, and mechanical properties. However, this study mainly analyzed total femur or femoral bone mass and did not focus on the proximal femur or a specific peri-hip muscle (Tang et al., 2022).

### 3.3 Exercise evidence

Exercise-intervention evidence provides a higher level of support for the question of whether this local unit can be modified. Early evidence regarding exercise and proximal femoral BMD or geometry was inconsistent. Kelley et al. analyzed individual patient data from 10 controlled studies involving 595 participants and did not observe a significant effect of exercise on FN BMD (Kelley and Kelley, 2006). Later conclusions became more consistently positive after two changes: the number and size of trials increased substantially, and training protocols shifted from low-intensity, low-impact activity towards programs emphasizing load magnitude and strain rate. Hejazi et al. included 53 studies and 2,896 postmenopausal women aged over 60 years and found that exercise significantly improved FN and trochanteric BMD (Hejazi et al., 2022). Mohebbi et al. subsequently reported more stable findings in an updated meta-analysis of 80 trials and 5,581 participants, with SMDs of 0.27 for FN BMD and 0.41 for TH BMD (Sehmisch et al., 2009). O’Bryan et al. showed in a meta-analysis of resistance training in older adults that progressive resistance training (PRT) improved lower-limb strength and increased femur/hip bone mass by an average of 2.77%, with clearer skeletal benefit when weight-bearing or impact-loading components were included (O’Bryan et al., 2022). A recent systematic review and meta-analysis further suggested that exercise benefits at the proximal femur extend beyond aBMD and include significant improvement in FN cortical thickness (SMD 0.61), particularly after at least eight months of high-intensity resistance plus high-impact training (Fujii et al., 2026). This body of evidence indicates that hip skeletal benefit is not a generalized whole-body exercise effect, but is closely related to sufficiently strong, local, and sustained mechanical stimulation.

Several site-specific RCTs directly demonstrate proximal femoral plasticity in response to local loading. In the LIFTMOR trial, postmenopausal women with low bone mass completed eight months of HiRIT (>85% one-repetition maximum[1RM], twice weekly). FN BMD increased by 0.3% in the intervention group and decreased by 1.9% in controls, accompanied by improved FN cortical thickness and functional measures (Gala et al., 2001). The MEDEX-OP trial further showed that HiRIT improved TH trabecular volumetric BMC (vBMC) and vBMD, FN and TH total vBMC, and geometric strength indices such as section modulus; some FN and TH structural parameters improved more clearly when antiresorptive medication was combined with training (Kistler-Fischbacher et al., 2022). Unilateral intervention studies are particularly informative for the local muscle-bone axis. Hartley et al. assigned postmenopausal women to six months of multidirectional hopping on one leg only. In the exercised leg, FN BMD, BMC, and section modulus increased by 0.81%, 0.69%, and 3.18%, respectively, while the contralateral control leg declined; MRI did not show worsening knee osteoarthritis markers (Hartley et al., 2020). Allison et al. similarly found, in a 12-month unilateral high-impact intervention in older men, that the exercised leg gained FN BMD, BMC, and CSA, whereas the control leg declined (Allison et al., 2013). Three-dimensional QCT analysis by Allison et al. showed that these effects were not uniformly distributed: cortical mass surface density increased by 2.7% on average and endocortical trabecular density by 6.4%, with gains localized mainly to the anterior and posterior FN, trochanteric region, and subcapital femoral head (Allison et al., 2015). O’Rourke et al. reached a similar conclusion using pre- and post-exercise 3D-DXA and finite-element modelling: exercise-induced changes in FN strength were determined mainly by local BMD and geometric changes in the superior neck, inferior neck, and greater trochanter rather than by average changes across the whole proximal femur (O’Rourke et al., 2023). These studies provide relatively direct clinical imaging support for local muscle-local bone coupling: exercise responses are spatially heterogeneous before they appear as modest overall BMD increases.

### 3.4 Summary

The hip evidence supports three main conclusions. First, there is genuine outcome-relevant coupling between peri-hip muscles and the proximal femur, but this coupling is muscle-specific, region-specific, and time-dependent rather than a simple parallel phenomenon of muscle loss and bone loss. Second, for hard endpoints such as first fracture, refracture, and mortality, muscle quality, especially CT-derived muscle density or myosteatosis, is often closer to real risk than muscle area and can provide additional information beyond conventional aBMD. Third, exercise-induced hip benefit is not automatic but depends on sufficiently strong local mechanical stimulation. When training meets the requirements of high load, high loading rate, and site-specific loading, proximal femoral aBMD, cortical thickness, CSA, section modulus, and finite-element strength can improve.

Caution remains necessary. A 2024 systematic review including 36 studies and 7,860 patients with hip fracture found that perioperative muscle-mass assessment did not consistently provide additional prognostic value beyond muscle strength in multivariable models (Prowse et al., 2024). CT-derived gluteal muscle density should therefore be described as a promising site-specific biomarker rather than as a fully standardized routine clinical endpoint. Overall, when local muscle quality declines and load-transfer efficiency weakens, and when proximal femoral bone mass and geometric reserve are insufficient, the risks of first fracture, second fracture, and mortality rise. This indicates that mismatch between muscle capacity and skeletal structural reserve is not unique to the spine, but may be a general principle of LMBU degeneration. Animal and biomechanical studies do not yet prove that improvement of a specific gluteal muscle directly enhances proximal femoral microstructure. They do, however, collectively show that local muscle loading, muscle disuse, hip abductor strength during fall impact, and exercise-induced FN strain can influence femoral bone mass, bone strength, or FN stress distribution. Direct evidence that exercise-induced improvement in a specific peri-hip muscle, such as the gluteus medius/minimus, gluteus maximus, or iliopsoas, causes adjacent proximal femoral microstructural adaptation remains limited. The peri-hip muscle-proximal femur unit is therefore best understood as an outcome-relevant and exercise-responsive local functional framework, with muscle-specific causality requiring further longitudinal trials integrating quantitative muscle imaging, QCT or 3D-DXA femoral assessment, biomechanical modelling, molecular markers, and fracture outcomes (**Table 2**).

**Table 2:** Evidence table for the peri-hip muscle-proximal femur local muscle-bone unit.

Author	Study type	Population/model	Muscle-bone assessment target	Methods	Main findings	Ref.
Ahedi et al. 2014	Cross-sectional study	321 participants from the Tasmanian Older Adult Cohort	Hip flexors, selected short external rotators, gluteus maximus and piriformis; TH/FN BMD	Association analysis of muscle CSA/strength and DXA-derived hip BMD	Hip flexors and selected short external rotators were mainly associated with TH and FN BMD, whereas gluteus maximus and piriformis were not significantly associated with BMD.	(Ahedi et al., 2014)
Pasco et al. 2015	Cross-sectional study	863 women	Hip flexor strength and hip abductor strength; TH BMD	Muscle-strength testing and DXA, with adjustment for total or appendicular lean mass	Hip flexor and hip abductor strength were positively associated with TH BMD; these associations disappeared after adjustment for total or appendicular lean mass.	(Pasco et al., 2015)
Capato et al. 2023	Cross-sectional study	94 independently living women aged >=60 years	Hip abductor peak torque; FN BMD and functional performance	Hip abductor peak torque, bone-density assessment and functional testing	Hip abductor peak torque was independently associated with FN BMD and multiple functional tests, supporting gluteus medius/hip abductor function as a local muscle-bone indicator.	(Capato et al., 2023)
Wang et al. 2020	Case-control CT study	438 patients with low-energy acute hip fracture and 316 healthy controls	Gluteal muscle area and density; FN/TH aBMD	CT-derived muscle area/density, CT-XA-derived aBMD and fracture-discrimination models	Gluteus medius/minimus density discriminated first hip fracture better than FN and TH aBMD, with AUCs of 0.88 for FN fracture and 0.95 for intertrochanteric fracture.	(Wang et al., 2020a)
Huang et al. 2024	Cross-sectional study	554 older women with hip fracture	Gluteus medius/minimus and gluteus maximus size and density; fracture type; TH aBMD	CT measurement of femoral parameters and comparison of femoral neck fracture with trochanteric fracture	Overall muscle parameters were higher in the FNF group than in the TRF group; after adjustment for age, BMI and TH aBMD, several gluteal parameters remained associated with TRF, particularly in women younger than 80 years.	(Huang et al., 2024)
Ma et al. 2026	Retrospective CT study	Middle-aged and older patients with hip fracture	Radiodensity of peri-hip muscles, including gluteus maximus, gluteus medius/minimus and adductors; fracture type/severity	CT measurement of peri-hip muscle radiodensity and CSA, with analysis according to fracture type/severity	Higher muscle radiodensity was more often observed in undisplaced femoral neck fracture, whereas lower radiodensity was associated with displaced femoral neck fracture or intertrochanteric fracture.	(Ma et al., 2026)
Wang et al. 2022	Prospective cohort study	Patients after first hip fracture	Gluteal muscle density; contralateral second hip fracture; FN/TH aBMD	CT-derived muscle density and aBMD with prospective fracture-risk modelling	After first hip fracture, contralateral second hip fracture risk was independently associated not only with aBMD but also with local gluteal muscle density; gluteus medius/minimus density showed the strongest association and remained significant after adjustment for FN or TH aBMD.	(Wang et al., 2022)
Wang et al. 2024	Prospective cohort study	Follow-up population after first hip fracture	Gluteus maximus and gluteus medius/minimus density; FN aBMD; contralateral second hip fracture	Time-dependent risk prediction using bone density and muscle density	Prediction of second hip fracture was time-dependent: FN aBMD was the stronger predictor within the first year, whereas gluteus maximus and gluteus medius/minimus density were more informative from the second year onwards.	(Wang et al., 2024)
Wang et al. 2023	Prospective cohort study	459 patients with hip fracture	Gluteus maximus area/density and gluteus medius/minimus density; mortality outcomes	CT-derived muscle area/density and mortality analysis	Lower gluteus maximus area, lower gluteus maximus density and lower gluteus medius/minimus density were associated with higher mortality risk; one-year mortality was mainly related to muscle density, whereas mortality after two years and beyond was more closely related to gluteus maximus area.	(Wang et al., 2023)
Ge et al. 2023	Prospective cohort study	394 older patients with hip fracture	Trochanteric aBMD; mortality risk	Follow-up analysis of hip aBMD and mortality risk	Higher trochanteric aBMD was associated with lower mortality risk, indicating that bone-side information is also independently related to post-hip-fracture outcomes.	(Ge et al., 2023)
Peng et al. 1994	Animal experiment	Ovariectomised rats	Strength/bone mass of the femoral neck, tibia and humerus	OVX model plus exercise intervention; measurement of bone strength and bone loss	OVX reduced the maximum breaking load of the femoral neck; exercise almost completely prevented the decline in femoral-neck mechanical strength and partially overcame trabecular bone loss. Individual peri-hip muscles were not specifically assessed.	(Peng et al., 1994)
Libouban et al. 2018	Animal experiment	Mouse unilateral quadriceps-paralysis model	Quadriceps atrophy; distal femur and tibial bone mass	Botulinum-toxin injection into the quadriceps and longitudinal micro-CT assessment of muscle and bone wasting	Paralysed-side muscle atrophy and ipsilateral trabecular bone loss changed in parallel and partly recovered several months later, suggesting that local muscle disuse is sufficient to drive ipsilateral bone loss.	(Libouban et al., 2018)
Tang et al. 2022	Animal exercise intervention	Ovariectomised rats	Femoral BMD, trabecular microarchitecture and mechanical properties; MSTN-related signalling	Weight-bearing treadmill training with assessment of bone mass, microarchitecture, mechanics and MSTN	Weight-bearing treadmill training increased femoral BMD and improved trabecular microarchitecture and mechanical properties. The study mainly analysed whole-femur or overall femoral bone mass, rather than the proximal femur or a specific peri-hip muscle.	(Tang et al., 2022)
Kelley et al. 2006	Individual-patient-data meta-analysis	10 controlled studies involving 595 postmenopausal women	Femoral neck BMD	Individual-patient-data meta-analysis of controlled clinical trials	No significant effect of exercise on FN BMD was observed, suggesting that early exercise evidence for FN BMD benefit was inconsistent.	(Kelley and Kelley, 2006)
Hejazi et al. 2022	Systematic review and meta-analysis	53 RCTs including 2,896 postmenopausal women aged >=60 years	Femoral neck and trochanteric BMD	Systematic review/meta-analysis of exercise-intervention RCTs	Exercise significantly improved femoral neck and trochanteric BMD, supporting an overall protective effect of exercise at key hip skeletal sites.	(Hejazi et al., 2022)
O’Bryan et al. 2022	Systematic review and meta-analysis	Exercise-training studies in older adults	Muscle strength and femur/hip bone mass/BMD	Meta-analysis of progressive resistance training	Progressive resistance training improved lower-limb strength and increased femur/hip bone mass by an average of 2.77%; skeletal benefit was clearer when weight-bearing or impact-loading components were included.	(O’Bryan et al., 2022)
Fujii et al. 2026	Systematic review and meta-analysis	Exercise-intervention studies in women	Proximal femoral cortical bone, especially FN cortical thickness	Systematic review/meta-analysis of exercise effects on proximal femoral cortical-bone indices	Exercise benefits were not limited to aBMD but also included improvement in FN cortical thickness, particularly after at least eight months of high-intensity resistance plus high-impact training.	(Fujii et al., 2026)
Kistler-Fischbacher et al. 2022	Randomised controlled trial (MEDEX-OP)	Postmenopausal women with or without bone medication	TH/FN vBMC, vBMD and geometric strength indices, including section modulus	HiRIT assessed using 3D-DXA and geometric strength indices	HiRIT improved TH trabecular vBMC/vBMD and FN/TH total vBMC and section modulus; some structural parameters appeared to improve more clearly when training was combined with antiresorptive medication.	(Kistler-Fischbacher et al., 2022)
Hartley et al. 2020	Randomised unilateral intervention trial	Postmenopausal women	Exercised and contralateral FN BMD, BMC and section modulus; knee OA imaging markers	Six-month unilateral multidirectional hopping intervention with DXA/MRI assessment	In the exercised leg, FN BMD, BMC and section modulus increased by approximately 0.81%, 0.69% and 3.18%, respectively, while the contralateral leg declined; MRI showed no worsening of knee OA imaging markers.	(Hartley et al., 2020)
Allison et al. 2013	Randomised unilateral intervention trial	Older men	Exercised and contralateral FN BMD, BMC and CSA	Twelve-month unilateral high-impact exercise, comparing the exercised and contralateral legs	The exercised leg gained FN BMD, BMC and CSA, whereas the contralateral leg declined, supporting a site-specific proximal-femoral response to local impact loading.	(Allison et al., 2013)
Allison et al. 2015	3D-QCT analysis of a randomised unilateral intervention	Older men	Proximal femoral density and endocortical trabecular density	Three-dimensional QCT analysis of bone mineral distribution after high-impact exercise	The exercise response was spatially non-uniform within the proximal femur; cortical mass surface density increased by 2.7% and endocortical trabecular density by 6.4%, with gains mainly localised to the anterior and posterior femoral neck, trochanteric region and subcapital femoral head.	(Allison et al., 2015)
O’Rourke et al. 2023	Pre-post exercise modelling study	Exercise-intervention participants	FN strength, local BMD and geometry	3D-DXA with finite-element/biomechanical modelling	Exercise-induced changes in FN strength were determined mainly by local BMD and geometric changes in the superior neck, inferior neck and greater trochanter, rather than by average changes across the whole proximal femur.	(O’Rourke et al., 2023)
Prowse et al. 2024	Systematic review	36 studies including 7,860 patients with hip fracture	Perioperative muscle mass/strength measurements and subsequent outcomes	Systematic review evaluating feasibility, acceptability and prognostic value	In patients with hip fracture, muscle-mass assessment did not consistently provide additional prognostic value beyond muscle strength in multivariable models; muscle imaging should therefore be regarded as a promising but not yet fully standardised adjunctive indicator.	(Prowse et al., 2024)

BMD, bone mineral density; aBMD, areal bone mineral density; BMC, bone mineral content; FN, femoral neck; FNF, femoral neck fracture; TH, total hip; TRF, trochanteric fracture; CSA, cross-sectional area; CT, computed tomography; DXA, dual-energy X-ray absorptiometry; QCT, quantitative computed tomography; 3D-DXA, three-dimensional dual-energy X-ray absorptiometry; vBMC/vBMD, volumetric bone mineral content/volumetric bone mineral density; HiRIT, high-intensity resistance and impact training; 1RM, one-repetition maximum; OVX, ovariectomised; OA, osteoarthritis; AUC, area under the curve; MSTN, myostatin; SMD, standardised mean difference; RCT, randomised controlled trial; meta-analysis, meta-analysis.

## 4 Exercise-responsive muscle-bone crosstalk mediated by mechanotransduction and secretome signalling

Within the LMBU, exercise should not be considered merely an increase in external load. It is more accurately understood as a set of mechanical cues generated by muscle contraction and characterized by specific temporal and spatial features. Turner and Robling noted that the osteogenic effects of exercise prescription depend strongly on loading properties, including strain magnitude, strain rate, number of loading cycles, and osteocyte sensitivity to mechanical stimulation (Turner and Robling, 2003). Robling et al. showed in a long-term rat ulna-loading model that, when the total number of loading cycles was the same, dividing daily loading into several short bouts with recovery intervals produced greater improvement in bone structure and mechanical properties than continuous loading. This suggests that osteocytes may desensitize rapidly during continuous loading and that intermittent recovery helps restore mechanosensitivity (Robling et al., 2002). Srinivasan et al. further showed that even low-magnitude loading close to that of daily activities can significantly enhance periosteal osteogenesis when brief rest intervals are inserted between loading cycles. Thus, the temporal structure of mechanical stimulation itself alters biological recognition of loading by bone (Srinivasan et al., 2002). New bone formation induced by mechanical loading is often localized to bone surfaces where strain change is greatest, indicating strong spatial directionality in bone adaptation (Robling et al., 2002).

These cues include strain magnitude, strain rate, loading frequency, intermittent rest time, and the anatomical distribution of muscle tensile and compressive forces (Robling et al., 2002; Srinivasan et al., 2002; Turner and Robling, 2003). Osteocytes, osteoblast-lineage cells, and BMMSCs sense these mechanical cues and translate them into intracellular calcium signalling, mechanosensitive transcriptional programs, and local secretome responses. Together, these responses coordinate bone formation, bone resorption, and marrow-cell lineage differentiation (Thompson et al., 2012; Lewis et al., 2017; Uda et al., 2017). This framework is particularly important in OS, because reduced muscle strength, fatty infiltration, and lower contractile loading may together impair skeletal mechanosensitivity and disrupt biochemical communication between muscle and bone (Sen et al., 2008; Uda et al., 2017; Chiapparelli et al., 2022).

For the local muscle-bone unit, exercise should therefore be understood as a muscle-contraction-derived mechanical cue that is translated within local tissues into intrinsic signalling pathways and ultimately drives site-specific bone remodeling (Turner and Robling, 2003; Thompson et al., 2012; Lewis et al., 2017). This concept is especially relevant to the lumbar spine and hip, where strain in the vertebral bodies and proximal femur is not uniformly distributed but arises mainly from regional tensile and compressive forces generated by the ES, MF, psoas, gluteus medius/minimus, gluteus maximus, and other peri-hip muscles (Lu et al., 2020; Chiapparelli et al., 2022; Li et al., 2022). Vertebral bodies contain abundant trabecular bone and may therefore be especially sensitive to reductions in mechanical loading caused by local muscle weakness or deterioration in muscle quality. Consistent with this, MRI-QCT studies show segment-specific associations between paraspinal CSA or fatty infiltration and lumbar vBMD (Zhao et al., 2019; Chiapparelli et al., 2022), while peri-hip muscle size and fatty infiltration are closely related to BMD in different subregions of the proximal femur (Lu et al., 2020; Li et al., 2022). This mechanism is especially relevant for vertebral trabecular bone, which has high metabolic activity and a large surface area and is therefore more susceptible to changes in local muscle force. Machireddy et al. used an ex vivo bioreactor-loading model of porcine vertebral trabecular bone and RNA sequencing of osteocytes under controlled mechanical loading. Differential gene expression was observed after one and three days of loading, involving inflammation, nuclear factor kappa-B (NF-κB)/tumor necrosis factor (TNF) signalling, and osteocyte regulatory networks. These findings suggest that osteocytes in vertebral-like trabecular bone display dynamic transcriptional responses to sustained mechanical stimulation, although coupling with the myogenic secretome remains to be verified (Machireddy et al., 2024).

In OS, exercise loading should be interpreted as mechanical signal input within the LMBU. Its core biological processes include mechanotransduction, osteoblast-osteoclast coupling, Wnt/β-catenin activation, sclerostin suppression, and integration of muscle-derived and bone-derived secretome signals. Choi et al. reviewed how osteocytes sense mechanical stimulation through the lacunar-canalicular network and regulate the dynamic balance between osteoblasts and osteoclasts (Choi et al., 2022). In a three-dimensional compression model using MLO-Y4 osteocyte-like cells, Zarka et al. showed that mechanical loading induced expression of several chemokines, including M-csf and Cxcl family members, through YAP/TAZ. This suggests that mechanically stimulated osteocytes can influence osteoclasts, osteoblasts, and marrow cells through secreted factors (Zarka et al., 2021). Wang et al. showed that the Piezo1-YAP axis can regulate osteoblast-osteoclast crosstalk through type II and type IX collagen-related matrix signals, thereby suppressing osteoclast differentiation and bone resorption (Wang et al., 2020b). Hu et al. further proposed a Piezo1-YAP-CCN1/2-Src axis in which CCN1/2, as secreted matricellular proteins, participates in osteocyte dendrite formation and also transmits mechanical information into the local microenvironment (Hu et al., 2025). Thus, exercise-induced mechanical stimulation is not confined to individual cells; it reshapes the secretome of osteocytes and osteoblast-lineage cells, creating a local pro-osteogenic and anti-resorptive signalling environment. Osteocytes are therefore central nodes linking mechanical loading, local bone remodeling, and secretome signalling.

Piezo channels are among the most representative mechanosensitive ion channels in the initial sensing of mechanical signals. Zhou et al. used Piezo1 or Piezo1/2 conditional knockout mouse models, *in vitro* osteogenic induction assays, fluid shear stress stimulation, substrate-stiffness culture systems, Ca^2+^ imaging, and the Piezo1 agonist Yoda1 to demonstrate that Piezo1/2 act as important mechanosensitive ion channels in osteogenic-lineage cells. Their study showed that Piezo1/2-mediated Ca^2+^ influx activates calcineurin and subsequently promotes the dephosphorylation, nuclear translocation and coordinated transcriptional activity of NFATc1, YAP1 and β-catenin. Conversely, loss of Piezo1/2 impaired osteogenic differentiation, increased bone resorption and aggravated skeletal fragility. These findings provide important mechanistic evidence that exercise-related mechanical loading can be translated into osteogenic responses through Piezo-mediated mechanotransduction. However, because the evidence is primarily derived from animal and cellular models, it should not be interpreted as direct proof of a causal mechanism within human site-specific local muscle–bone units (Zhou et al., 2020). Piezo1 may be the dominant mechanosensitive channel in bone. Wang et al. used a Prx1-Cre-mediated Piezo1 conditional knockout model to show that Piezo1 regulates bone homeostasis in osteoblast-lineage cells and influences bone remodeling through osteoblast-osteoclast crosstalk, while Piezo2 may play a weaker or redundant role in bone (Wang et al., 2020b).

YAP/TAZ and β-catenin form central integrative modules that convert mechanical signals into osteogenic transcriptional programs. Zhou et al. showed that Piezo1/2 deletion reduced YAP1 and β-catenin activity, while activation of β-catenin with the GSK3 inhibitor BIO partially rescued skeletal defects in Piezo1/2-mutant mice, indicating that β-catenin is an important effector downstream of Piezo mechanotransduction (Zhou et al., 2020). In osteocyte-like cells, Zarka et al. established a three-dimensional collagen-gel compression model of MLO-Y4 cells and found that mechanical loading induced YAP/TAZ nuclear translocation and upregulated chemokines including M-csf, Cxcl1, Cxcl2, Cxcl3, Cxcl9, and Cxcl10. After YAP/TAZ knockdown, some mechanically responsive genes were attenuated, indicating that YAP/TAZ participates not only in osteogenic differentiation but also in the secretome remodeling of mechanically stimulated osteocyte-like cells (Zarka et al., 2021). Hu et al. further showed in a late osteoblast/osteocyte Piezo1-deficient model that Piezo1 loss reduced bone mass, impaired osteocyte maturation, and reduced dendrite number, length, and network formation. The mechanism involved the Piezo1-YAP-CCN1/2-Src axis, in which CCN1/2 as YAP downstream secreted factors activate Src, promote actin polymerization, and reinforce YAP activity through positive feedback (Hu et al., 2025).

Downstream of mechanotransduction, mechanical loading downregulates SOST expression or reduces sclerostin protein abundance, thereby relieving inhibition of Wnt/β-catenin signalling (Robling et al., 2008; Tu et al., 2012; Lyons et al., 2017; Williams et al., 2020). Sclerostin is secreted mainly by osteocytes and suppresses osteogenic activity by inhibiting low-density lipoprotein receptor-related protein 5/6 (LRP5/6)-mediated canonical Wnt signalling (Li et al., 2005; Poole et al., 2005). The traditional view is that mechanical loading reduces Sost transcription (Robling et al., 2008; Tu et al., 2012). More recent studies indicate that mechanical stimulation can also rapidly regulate sclerostin at the protein level (Lyons et al., 2017; Williams et al., 2020). Gould et al. showed in mouse models and rodent cell lines that mechanical loading and parathyroid hormone (PTH) can induce lysosomal degradation of sclerostin protein within minutes, indicating that sclerostin regulation by mechanical stimuli includes rapid post-translational degradation in addition to delayed transcriptional suppression (Gould et al., 2021). Buck et al. subsequently showed that nitric oxide (NO) contributes to rapid sclerostin protein loss after mechanical loading and acts downstream of Ca2+ influx and calcium/calmodulin-dependent protein kinase II(CaMKII) activation (Buck et al., 2024). Exercise may therefore rapidly reduce osteocytic sclerostin through a Ca^2+^/CaMKII/NO axis, relieving inhibition of Wnt/β-catenin signalling.

Wnt signalling itself is required for mechanically induced bone formation. Lawson et al. used an Osx-lineage Wnt1 conditional knockout model and tibial mechanical loading to show that Wnt1 induction in osteoblast-lineage cells is essential for the bone-formation response to loading. Deletion of Wnt1 in Osx-lineage cells markedly weakened the skeletal response to mechanical loading (Lawson et al., 2022). Ahmad et al. further explored downstream mechanisms of Wnt1 and found that mechanical stimulation induced Wnt1 expression in osteoblasts and upregulated Runx2 and Sp7; siRNA knockdown of Wnt1 inhibited these osteogenic responses. RNA sequencing identified Plat as an important Wnt1 downstream target, and Wnt1 regulated Plat expression through β-catenin activation to promote mechanically induced osteoblast differentiation (Ahmad et al., 2024). Thus, mechanical stimulation does not promote bone formation through physical compression alone. Instead, it establishes a pro-osteogenic microenvironment through SOST/sclerostin suppression, Wnt1 induction, and β-catenin transcriptional activation.

BMMSCs are also important mechanosensitive cells in the exercise-responsive LMBU because the balance between osteogenic and adipogenic differentiation directly determines bone formation and marrow fat accumulation. Wang et al. used BMMSC-specific Piezo1 deletion, *in vitro* osteogenic and adipogenic differentiation assays, and exercise-related *in vivo* models to show that Piezo1 in BMMSCs is a key mechanosensor that suppresses marrow adipogenesis and maintains bone strength (Wang et al., 2025). Piezo1-deficient BMMSCs preferentially underwent adipogenesis rather than osteogenesis, and mice developed OP, increased marrow adiposity, and resistance to the beneficial effects of exercise on bone mass and marrow fat. Mechanistically, Piezo1 deficiency enhanced autocrine activation of CCR2 by Ccl2 and induced Lcn2 through NF-κB to promote adipogenesis. Conversely, Piezo1 opening induced Klf2 via CaMKII, thereby suppressing c-Jun activation, Ccl2 production, and marrow adipogenesis, while promoting osteogenesis (Wang et al., 2025). This work links mechanosensing, inflammatory autocrine loops, and BMMSC fate decisions, providing an important explanation for how insufficient exercise or mechanical stimulation may promote BMMSC adipogenic bias, marrow fat accumulation, and reduced bone formation in OS.

**Figure 1:**
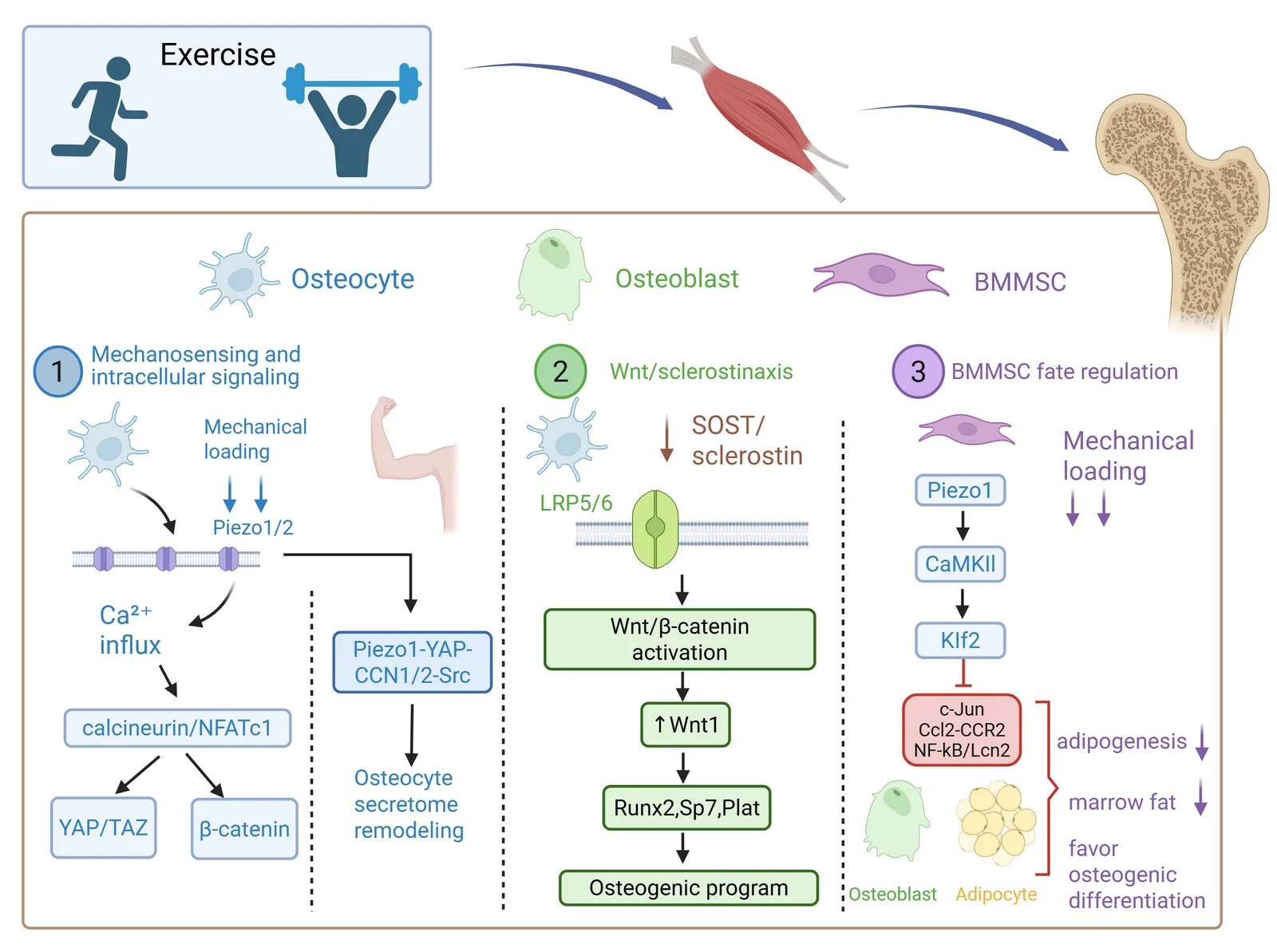
Explanation: Exercise generates mechanical loading through muscle contraction and transmits this loading to bone, driving adaptive remodelling of the LMBU. First, osteocytes sense mechanical stimulation through Piezo1/2, inducing Ca^2+^ influx and activating calcineurin/NFATc1, YAP/TAZ, and β-catenin signalling; the Piezo1-YAP-CCN1/2-Src axis also participates in bone-remodelling regulation. Second, mechanical stimulation reduces SOST/sclerostin levels, relieving inhibition of the LRP5/6-Wnt/β-catenin pathway. Mechanical stimulation can also induce osteoblastic Wnt1 expression, upregulate Runx2 and Sp7, and regulate Plat expression, thereby promoting mechanically induced osteoblast differentiation. Finally, Piezo1 deficiency enhances Ccl2-mediated autocrine activation of CCR2 and induces Lcn2 through NF-κB, promoting adipogenic differentiation. Conversely, Piezo1 opening induces Klf2 via CaMKII, suppresses c-Jun activation, Ccl2 production, and marrow adipogenesis, shifts cellular fate from adipogenesis towards osteogenesis, and reduces marrow fat deposition.

In summary, exercise loading first acts as a mechanical signal input within the LMBU and is sensed by osteocytes, osteoblast-lineage cells, and BMMSCs. Through Piezo-mediated Ca^2+^ influx and mechanosensitive transcriptional programs including calcineurin/NFAT, YAP/TAZ, and β-catenin, mechanical stimulation is translated into local bone-remodeling responses that promote osteogenesis, suppress resorption, and inhibit marrow adipogenesis. Exercise also reduces SOST transcription and sclerostin protein levels, relieving inhibition of Wnt1/β-catenin signalling. BMMSC adipogenic regulation, the CCN1/2-Src axis, chemokines, and other secreted signals further reshape the local microenvironment. However, most evidence for PIEZO1/2, SOST/sclerostin, Wnt1/β-catenin, and related pathways in muscle-bone communication comes from cellular experiments, long-bone loading models, disuse models, or genetically modified mice. Direct evidence that exercise-induced remodeling of a specific lumbar paraspinal or peri-hip muscle causes molecular changes in an adjacent vertebral body or proximal femur remains limited. The lumbar paraspinal-vertebral unit and the peri-hip muscle-proximal femur unit should therefore be described as anatomically plausible and mechanistically interpretable translational models, not as fully proven causal pathways. Future studies need to integrate targeted muscle-group training, quantitative muscle imaging, QCT or HR-pQCT bone-structure assessment, biomechanical modelling, and fracture or fall endpoints in a longitudinal framework to test causality and clinical modifiability (**Figure 1**).

### 4.1 Muscle-side secretome signals

Mechanotransduction explains how exercise is recognized by osteocytes, osteoblast-lineage cells, and BMMSCs, but it does not fully capture the systemic effects of exercise on muscle and bone. Mechanical loading provides site specificity, whereas secretome signals provide metabolic and inflammatory context. Exercise can reshape the endocrine and secretome states of both muscle and bone, coupling local mechanical stimulation to systemic metabolism, inflammation, bone remodeling, and muscle anabolism (Pedersen and Febbraio, 2012; Li et al., 2019; Severinsen and Pedersen, 2020; Leuchtmann et al., 2021). This is particularly relevant in OS, which involves not only parallel loss of muscle and bone, but also reduced contractile capacity, fatty muscle infiltration, insufficient bone formation, increased bone resorption, marrow fat accumulation, and low-grade chronic inflammation (Drey et al., 2016; Hirschfeld et al., 2017; Karsenty and Mera, 2018; Kirk et al., 2020). Exercise-responsive muscle-bone crosstalk should therefore be understood from both muscle-side and bone-side secretome directions.

Muscle-side secretome signals are an important component of muscle-bone communication. Myostatin is generally regarded as a negative myokine that inhibits muscle growth. In ageing, disuse, and SP, increased myostatin signalling can suppress muscle protein synthesis, promote muscle atrophy, and further weaken mechanical loading of bone by muscle. From a muscle-bone crosstalk perspective, myostatin does not act on muscle alone. Experimental evidence shows that myostatin can alter the osteocyte secretome, suppress osteocyte-derived exosomal miR-218, and accompany increased SOST, DKK1, and RANKL-related signalling, thereby creating a microenvironment that inhibits Wnt-mediated osteogenesis and promotes bone resorption (Qin et al., 2017). In OS, myostatin may therefore be understood as a negative muscle-derived signal linking muscle catabolism to suppressed bone formation. For the LMBU, direct human evidence remains lacking on whether degenerated paraspinal or peri-hip muscles can influence adjacent vertebrae or proximal femora through elevated local myostatin concentrations.

Irisin is an exercise-related myokine generated by cleavage of fibronectin type III domain-containing protein 5 (FNDC5), often associated with peroxisome proliferator-activated receptor gamma coactivator 1 alpha(PGC-1α)-mediated exercise adaptation. Animal studies show that recombinant irisin increases cortical bone mass and strength, promotes osteoblast-mediated bone formation, and regulates osteocytic sclerostin expression (Colaianni et al., 2015). Later work suggested that αV-class integrins, especially αVβ5, may act as functional irisin receptors in osteocytes and adipocytes (Kim et al., 2018). In human studies, plasma irisin levels increased after aerobic interval training compared with sedentary conditions, indicating exercise responsiveness. Targeted mass spectrometry has confirmed the presence of circulating irisin and its association with exercise, although assay methods, exercise protocols, and physiological dose ranges remain debated (Jedrychowski et al., 2015). Irisin therefore represents a potentially osteoanabolic exercise-induced myokine that may participate in muscle-bone crosstalk through osteocyte and osteoblast pathways. Its dose-response relationship, measurement standardization, and direct causal effects on bone mass, muscle function, and the LMBU in OS remain to be established. In local units, irisin is more likely to act as a systemic exercise-adaptation signal that modulates skeletal mechanosensitivity than as a proven paraspinal-vertebral or peri-hip-proximal femoral local factor.

Insulin-like growth factor 1 (IGF-1) is a classic anabolic factor shared by muscle and bone. In skeletal muscle, IGF-1 and its mechanosensitive splice variants participate in resistance-training-induced protein synthesis, satellite-cell activation, and fiber hypertrophy. In bone, IGF-1 promotes osteoblast differentiation, matrix synthesis, and bone formation (Bikle et al., 2015). IGF-1 stored in the bone matrix can be released during bone remodeling and can promote mesenchymal stem/stromal-cell osteogenic differentiation through mechanistic target of rapamycin (mTOR) signalling, thereby maintaining adult bone mass (Xian et al., 2012). In OS, reduced IGF-1 signalling may simultaneously impair muscle anabolism and bone formation, lowering adaptive capacity in response to exercise. Progressive resistance training or mechanical loading can regulate local skeletal-muscle expression of IGF-1 and mechano-growth factor (MGF), and intraosseous IGF-1 signalling contributes to mechanically induced bone formation (Bamman et al., 2001). Targeted paraspinal or peri-hip training may therefore enhance local mechanical loading while improving an IGF-1-related anabolic background, jointly supporting adjacent bone adaptation. However, direct evidence that muscle-derived IGF-1 alone mediates structural improvement in specific paraspinal-vertebral or peri-hip-proximal femoral units is still lacking.

Interleukin-6 (IL-6) is persistently elevated in chronic low-grade inflammation and is often associated with muscle catabolism, increased bone resorption, and frailty. In contrast, pulsatile IL-6 released by contracting skeletal muscle during acute exercise has a different physiological meaning. Evidence shows that circulating IL-6 during exercise is largely muscle-derived, and that myogenic IL-6 can act on osteoblasts to promote release of bioactive osteocalcin. Osteocalcin then feeds back on myofibers to enhance nutrient uptake and catabolic capacity, supporting exercise endurance and adaptation (Chowdhury et al., 2020). IL-6 therefore has dual properties as both an acute exercise myokine and a chronic inflammatory mediator. In OS, regular exercise may restore physiological pulsatile IL-6 release and the IL-6/osteocalcin feedback loop, improving muscle metabolism and bone endocrine function. Conversely, chronically elevated inflammatory IL-6 may drive muscle atrophy and bone loss.

Beta-aminoisobutyric acid (BAIBA), particularly L-BAIBA, is an exercise-induced muscle-derived small-molecule metabolite and can be considered an exerkine. Experimental studies show that L-BAIBA protects osteocytes from reactive oxygen species-induced cell death through the MAS-related G protein-coupled receptor D (MRGPRD) receptor and reduces both bone loss and muscle-function decline in hindlimb-suspension disuse models (Kitase et al., 2018). BAIBA is therefore particularly relevant to the connection between disuse-induced bone loss and muscle dysfunction. Its circulating levels, receptor expression, and exercise dose-response relationships in older adults with OS require further human investigation.

Apelin is an emerging exercise-related myokine. Muscle contraction can induce apelin production, and apelin levels decline with ageing. In animal models, apelin improves age-related SP, enhances muscle metabolism, mitochondrial function, and muscle stem-cell function (Vinel et al., 2018). Bone-side studies also suggest that apelin-13 promotes osteogenic differentiation and mineralization of human BMMSCs, partly through Wnt/β-catenin signalling (Hang et al., 2019). The effects of apelin in bone may depend on cell type, disease state, and microenvironment. Apelin can be discussed as an emerging exercise myokine, but should not be presented as an established clinical anti-osteoporotic target. Its potential to link exercise-induced improvements in muscle metabolism with recovery of marrow osteogenic potential remains to be verified.

Interleukin-33 (IL-33) is a newer muscle-derived immunomodulatory factor that may provide an immune-endocrine explanation for the muscle atrophy-bone loss axis. Lower circulating IL-33 levels in older adults have been associated with the coexistence of SP and OP. In animal models, weight-bearing exercise or muscle strengthening increases muscle IL-33 and improves bone formation, whereas botulinum toxin-induced muscle atrophy has the opposite effect. Mechanistically, muscle-derived IL-33 can act through the ST2 receptor on CD8+ T cells, promote CCL5 release, and thereby regulate bone metabolism and bone remodeling (Liu et al., 2025). This finding provides a new muscle-immune cell-bone remodeling pathway for exercise-induced muscle-bone crosstalk.

Ma et al. reported in Cell Metabolism that skeletal muscle can secrete muscle-derived extracellular vesicles (Mu-EVs). These Mu-EVs can reach bone tissue through the circulation and be taken up by BMMSCs. Proteomic and functional experiments showed that Mu-EVs can carry glycolytic proteins, including lactate dehydrogenase A, enhance glycolysis in BMSCs, promote osteogenic differentiation, and protect against disuse-induced OP in mice (Ma et al., 2023). Skeletal muscle therefore contributes to bone regulation not only by providing mechanical stimulation, but also through EVs and myogenic secreted factors that influence marrow stromal-cell metabolism and osteogenic fate. It has not yet been shown that EVs from a specific local muscle group act specifically on an adjacent vertebral body or proximal femur. Human exercise-intervention studies are needed to verify their biomarker value. Nevertheless, the EV mechanism provides an important direction for understanding how exercise regulates the marrow microenvironment beyond mechanical loading.

### 4.2 Bone-side secretome signals

Bone-side secretome signals also play important roles, including osteocalcin, sclerostin, RANKL/osteoprotegerin (OPG), fibroblast growth factor 23 (FGF23), prostaglandin E2 (PGE2), and transforming growth factor beta (TGF-β). Osteocalcin is a classic bone-matrix protein secreted by osteoblasts and one of the most representative bone-derived endocrine factors. Animal and mechanistic studies show that exercise promotes bioactive osteocalcin release, and that osteocalcin acts on myofibers to enhance glucose and fatty-acid uptake and catabolism, supporting optimal exercise adaptation (Mera et al., 2016a). Osteocalcin signalling is also important for maintaining muscle mass in older mice, and its decline may contribute to age-related loss of muscle function (Mera et al., 2016b). In animal models, exercise first activates bone remodeling through muscle contraction and mechanical loading; bone then feeds back through osteocalcin to enhance muscle metabolic capacity, forming a muscle-bone-muscle positive loop. Human studies in postmenopausal women with OP show that osteocalcin forms are associated with skeletal muscle mass, muscle function, and fall risk (Vitale et al., 2021). For OS, impairment of this mechanism may contribute to reduced exercise tolerance, insufficient muscle anabolism, and inadequate bone-formation response. Osteocalcin should, however, be regarded mainly as a systemic bone endocrine signal; evidence for vertebral or proximal femoral site specificity is lacking.

Sclerostin, secreted mainly by osteocytes, is an important inhibitor of Wnt/β-catenin osteogenic signalling and is more closely linked to local paracrine regulation, although circulating levels are often used as indicators of osteocyte status. Mechanical loading and exercise can reduce SOST expression or promote rapid degradation of sclerostin protein, relieving inhibition of LRP5/6-mediated Wnt signalling and enhancing osteogenesis (Gould et al., 2021; Buck et al., 2024). In OS, reduced muscle force and insufficient local mechanical stimulation may result in inadequate suppression of osteocytic sclerostin, leaving local bone formation in a low-response state. Sclerostin is one of the most suitable bone-side factors for discussion within the LMBU because its expression is directly regulated by local strain and fluid shear stress. If targeted paraspinal or peri-hip training generates sufficient mechanical strain in adjacent vertebral bodies or the proximal femur, local sclerostin reduction could theoretically enhance Wnt-mediated osteogenesis at that site. However, direct human evidence for local sclerostin changes in the paraspinal-vertebral or peri-hip-proximal femoral axis remains insufficient.

RANKL and OPG are key bone-derived regulators of osteoclastogenesis and bone resorption. Exercise and mechanical loading are generally thought to suppress bone resorption by reducing pro-osteoclastogenic signalling and improving the OPG/RANKL balance, but clinical studies of circulating markers are heterogeneous. Serum RANKL, OPG, or the OPG/RANKL ratio cannot be equated directly with local bone remodeling status (Chi et al., 2023). RANKL/OPG is therefore better understood as a local output of osteoblast-osteoclast coupling in adjacent bone tissue rather than as a purely systemic endocrine factor.

FGF23 is a bone-derived endocrine hormone produced mainly by osteocytes and osteoblasts. Its core functions are regulation of renal phosphate excretion and vitamin D metabolism. From an OS perspective, abnormally elevated FGF23 is associated with chronic kidney disease, mineral-metabolism disorders, bone disease, and impaired muscle function. However, the relationships between exercise, mineral metabolism, BAIBA, and osteocyte status are complex, and it remains unclear whether FGF23 is a major exercise-responsive muscle-bone mediator in ordinary age-related OS (Sirikul et al., 2022).

PGE2 is an important local lipid mediator of osteocyte mechanosensitivity. Mechanical loading promotes PGE2 release through osteocytic connexin 43 (Cx43) hemichannels, enhances bone formation, and reduces sclerostin expression (Zhao et al., 2022). More importantly, osteocytic Cx43 hemichannels may participate in bone-to-muscle feedback. Experimental studies show that inhibition of osteocytic Cx43 hemichannels reduces fast-twitch muscle-fiber mass and muscle function, whereas PGE2 supplementation partially improves the muscle phenotype (Li et al., 2021). This suggests that osteocytes are not only intraosseous mechanosensors, but may also influence muscle quality through secreted mediators such as PGE2. Because PGE2 has a short half-life and relatively local action, it is theoretically closer to the paracrine regulatory model of the LMBU than systemic factors such as osteocalcin. Current evidence, however, derives mainly from animal and cellular studies and does not directly prove that the same mechanism operates in the human paraspinal-vertebral or peri-hip-proximal femoral axis.

TGF-β is stored abundantly in the bone matrix and can be released during bone resorption. Physiological levels participate in bone remodeling and tissue repair, but pathological over-release may adversely affect muscle. In bone-metastasis models, TGF-β released during bone destruction causes muscle weakness through the NOX4/RyR1 oxidative-stress pathway (Waning et al., 2015). Although this evidence comes primarily from tumor-associated bone disease rather than ordinary OS or exercise-intervention studies, it suggests that increased bone resorption, bone-matrix factor release, and impaired muscle function may form a pathological bone-to-muscle feedback pathway.

**Figure 2:**
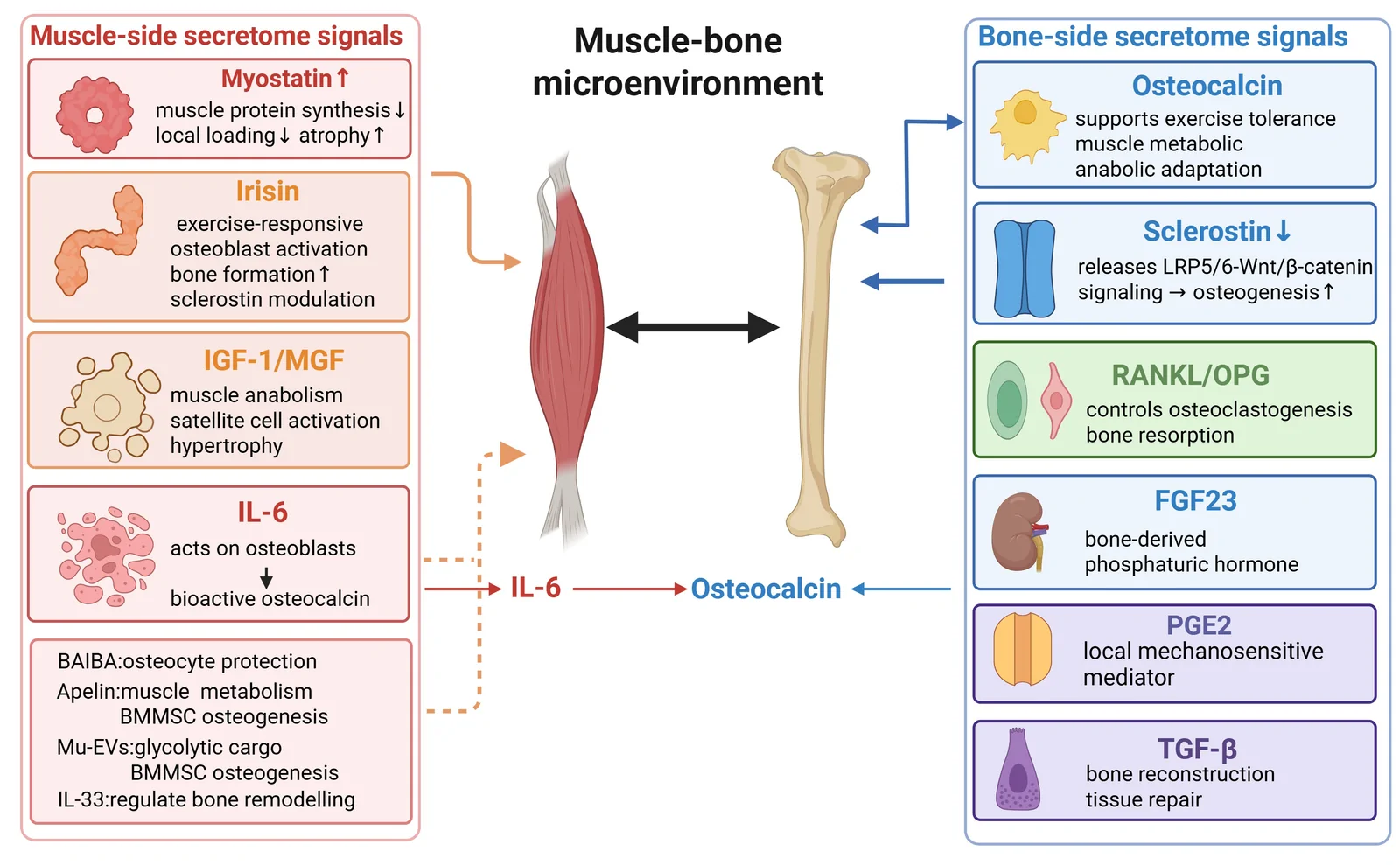
Explanation: Muscle and bone communicate through secreted signals. Myostatin is predominantly negative, inhibiting muscle protein synthesis, promoting muscle atrophy, and weakening local loading. Irisin is an exercise-induced myokine with potential osteoanabolic effects and may participate in muscle-bone crosstalk through osteocyte- and osteoblast-related pathways. Skeletal-muscle IGF-1 and its mechanosensitive splice variants participate in protein synthesis, satellite-cell activation, and fibre hypertrophy after resistance training; progressive resistance training or mechanical loading can regulate local skeletal-muscle IGF-1/MGF expression. During exercise, IL-6 can act on osteoblasts to promote release of bioactive osteocalcin. BAIBA protects osteocytes from reactive oxygen species-induced death; apelin promotes osteogenic differentiation and mineralisation of human BMMSCs; Mu-EVs deliver glycolytic cargo to BMSCs, enhance BMSC glycolysis and osteogenic differentiation, and protect against disuse-induced bone loss; and IL-33 provides a new muscle-immune cell-bone remodelling pathway for exercise-induced muscle-bone crosstalk. Osteocalcin supports exercise tolerance and muscle metabolic and anabolic adaptation. Sclerostin reduction releases LRP5/6-mediated Wnt/β-catenin signalling and enhances osteogenesis. RANKL/OPG regulates osteoclastogenesis. FGF23 is a bone-derived phosphaturic hormone. PGE2 is a local mechanosensitive mediator. TGF-β is related to bone remodelling and tissue repair.

From the perspective of the LMBU, exercise-responsive endocrine and secretome signalling should not be interpreted as evidence that each local muscle group directly targets its adjacent skeletal site through a unique secreted factor. A model more consistent with current evidence is that local muscle contraction first determines the mechanical cues received by adjacent bone, while muscle-derived and bone-derived secreted factors regulate the ability of that local bone tissue to respond to those cues. In other words, mechanical loading provides site specificity, whereas secretome signalling provides metabolic, inflammatory, and cell-fate context. When paraspinal or peri-hip muscles degenerate, local mechanical input decreases. At the same time, elevated myostatin, insufficient IGF-1 or apelin, weakened exercise-related IL-6/osteocalcin loops, abnormal EV cargo, and chronic inflammation may collectively reduce the osteogenic response of adjacent vertebrae or the proximal femur. Conversely, progressive resistance training, impact training, or weight-bearing training that reaches an adequate mechanical threshold may promote local bone adaptation through sclerostin suppression, osteocalcin release, improved OPG/RANKL balance, PGE2 release, and enhanced BMMSC osteogenic metabolism (**Figure 2**).

## 5 Conclusion

OS should be understood as the result of a combined disturbance within local muscle-bone functional units, involving muscle contractile loading, skeletal mechanosensitivity, marrow-cell fate, and muscle-derived and bone-derived secretome signals. The significance of exercise is not limited to increasing systemic BMD or muscle mass. Rather, exercise influences the adaptive remodeling of LMBUs, including the lumbar paraspinal muscle-vertebral body unit and the peri-hip muscle-proximal femur unit, through site-specific mechanical input and modulation of the secretome background. Based on the LMBU framework, this review integrates imaging, animal, exercise-intervention, and molecular-mechanistic evidence for the lumbar paraspinal-vertebral and peri-hip-proximal femoral units, and argues that muscle-bone crosstalk in OS is anatomically site-specific and exercise-responsive.

At the lumbar spine, paraspinal muscle-quality indices such as fatty infiltration, elevated PDFF, and reduced FCSA are associated with lower lumbar BMD or vBMD, vertebral fracture, and refracture risk. Animal disuse models and exercise-intervention studies support the possibility that altered local muscle loading can affect adjacent vertebral adaptation, but human studies have not yet shown that imaging improvement in a specific paraspinal muscle directly causes enhanced adjacent vertebral strength. At the hip, peri-hip muscle quality is associated with FN or intertrochanteric fracture phenotype, contralateral refracture, and mortality. High-load, high-rate, and site-specific exercise can improve proximal femoral aBMD, cortical thickness, CSA, section modulus, and finite-element strength. Nevertheless, there is still insufficient evidence that improvement of a specific gluteal muscle or the iliopsoas directly causes proximal femoral microstructural adaptation.

Mechanistically, exercise is best conceptualized as a set of regional mechanical cues generated by muscle contraction. Osteocytes, osteoblast-lineage cells, and BMMSCs convert these cues into osteogenic, anti-resorptive, and anti-adipogenic responses through Piezo, Ca^2+^/calcineurin/NFAT, YAP/TAZ, β-catenin, Wnt1, and SOST/sclerostin pathways. However, most mechanistic evidence comes from cells, animals, long-bone loading models, or genetically modified models and cannot be equated directly with local human causal pathways in the lumbar spine or hip.

Overall, the exercise-responsive LMBU provides a coherent and clinically relevant framework for understanding OS. Rather than viewing muscle degeneration and bone loss as independent processes, this framework emphasizes their coordinated disruption through impaired mechanical loading, altered mechanotransduction, dysregulated marrow-cell fate, and abnormal muscle- and bone-derived secretome signalling. Direct evidence linking local muscle improvement, adjacent bone structural adaptation, secretome remodeling, and reduced falls or fracture risk within a single longitudinal design is still lacking. Nevertheless, current evidence supports the LMBU as an important direction for risk stratification and optimization of exercise intervention in OS. Future multi-modal, mechanism-guided RCTs and longitudinal cohorts should incorporate quantitative muscle imaging, QCT or HR-pQCT bone-structure assessment, biomechanical modelling, secretome and EV biomarkers, functional outcomes, and hard fracture endpoints within the same research framework. This will help move OS management from traditional empirical rehabilitation towards mechanism-guided precision exercise intervention.

## Glossary

OS: Osteosarcopenia
OP: osteoporosis
SP: sarcopenia
LMBU: local muscle-bone unit
BMD: bone mineral density
aBMD: areal bone mineral density
vBMD: volumetric bone mineral density
BMC: bone mineral content
vBMC: volumetric bone mineral content
CSA: cross-sectional area
FCSA: functional cross-sectional area
PDFF: proton density fat fraction
MRI: magnetic resonance imaging
CT: computed tomography
QCT: quantitative computed tomography
HR-pQCT: high-resolution peripheral quantitative computed tomography
DXA: dual-energy X-ray absorptiometry
3D-DXA: three-dimensional dual-energy X-ray absorptiometry
DECT: dual-energy computed tomography
HU: Hounsfield units
BMI: body mass index
RCT: randomized controlled trial
HiRIT: high-intensity resistance and impact training
PRT: progressive resistance training
1RM: one-repetition maximum
OVX: ovariectomised
SD: Sprague-Dawley
FN: femoral neck
TH: total hip
FNF: femoral neck fracture
TRF: trochanteric fracture
MF: multifidus
ES: erector spinae
IMAT: intermuscular adipose tissue
SMI: skeletal muscle mass index
PVP: percutaneous vertebroplasty
PKP: percutaneous kyphoplasty
AUC: area under the curve
SMD: standardized mean difference
BMMSC: bone marrow mesenchymal stem/stromal cell
BMMSCs: bone marrow mesenchymal stem/stromal cells
BMSC: bone marrow stromal cell
BMSCs: bone marrow stromal cells
EVs: extracellular vesicles
Mu-EVs: muscle-derived extracellular vesicles
RANKL: receptor activator of nuclear factor kappa-B ligand
OPG: osteoprotegerin
NFATc1: nuclear factor of activated T cells c1
NF-κB: nuclear factor kappa-B
TNF: tumor necrosis factor
PPARγ: peroxisome proliferator-activated receptor gamma
GSK-3β: glycogen synthase kinase-3 beta
PI3K: phosphoinositide 3-kinase
IL-1β: interleukin-1 beta
IL-6: interleukin-6
IL-33: interleukin-33
IGF-1: insulin-like growth factor 1
MGF: mechano-growth factor
mTOR: mechanistic target of rapamycin
BAIBA: beta-aminoisobutyric acid
L-BAIBA: L-beta-aminoisobutyric acid
MRGPRD: MAS-related G protein-coupled receptor D
FNDC5: fibronectin type III domain-containing protein 5
PGC-1α: peroxisome proliferator-activated receptor gamma coactivator 1 alpha
FGF23: fibroblast growth factor 23
PGE2: prostaglandin E2
TGF-β: transforming growth factor beta
Cx43: connexin 43
PTH: parathyroid hormone
NO: nitric oxide
CaMKII: calcium/calmodulin-dependent protein kinase II

## References

[B43] Ahedi H. Aitken D. Scott D. Blizzard L. Cicuttini F. Jones G. (2014). The association between hip muscle cross-sectional area, muscle strength, and bone mineral density. Calcif. Tissue Int. 95, 64–72. doi: 10.1007/s00223-014-9863-6 24829114

[B90] Ahmad M. Haffner-Luntzer M. Schoppa A. Najafova Z. Lukic T. Alexander Y. T. . (2024). Mechanical induction of osteoanabolic Wnt1 promotes osteoblast differentiation via Plat. FASEB J. 38(4), e23489. doi: 10.1096/fj.202301424RR 38407813

[B32] Aliprantis A. O. Stolina M. Kostenuik P. J. Poliachik S. L. Warner S. E. Bain S. D. . (2012). Transient muscle paralysis degrades bone via rapid osteoclastogenesis. FASEB J. 26, 1110–1118. doi: 10.1096/fj.11-196642 22125315 PMC3289507

[B62] Allison S. J. Folland J. P. Rennie W. J. Summers G. D. Brooke-Wavell K . (2013). High impact exercise increased femoral neck bone mineral density in older men: a randomised unilateral intervention. Bone 53, 321–328. doi: 10.1016/j.bone.2012.12.045 23291565

[B63] Allison S. J. Poole K. E. S. Treece G. M. Gee A. H. Tonkin C. Rennie W. J. . (2015). The influence of high-impact exercise on cortical and trabecular bone mineral content and 3D distribution across the proximal femur in older men: a randomized controlled unilateral intervention. J. Bone Miner. Res. 30, 1709–1716. doi: 10.1002/jbmr.2499 25753495

[B106] Bamman M. M. Shipp J. R. Jiang J. Gower B. A. Hunter G. R. Goodman A. . (2001). Mechanical load increases muscle IGF-I and androgen receptor mRNA concentrations in humans. Am. J. Physiol. Endocrinol. Metab. 280, E383–E390. doi: 10.1152/ajpendo.2001.280.3.E383 11171591

[B104] Bikle D. D. Tahimic C. Chang W. Wang Y. Philippou A. Barton E. R. (2015). Role of IGF-I signaling in muscle bone interactions. Bone 80, 79–88. doi: 10.1016/j.bone.2015.04.036 26453498 PMC4600536

[B9] Bonewald L. F. Kiel D. P. Clemens T. L. Esser K. Orwoll E. S. O’Keefe R. J. . (2013). Forum on bone and skeletal muscle interactions: summary of the proceedings of an ASBMR workshop. J. Bone Miner. Res. 28, 1857–1865. doi: 10.1002/jbmr.1980 23671010 PMC3749267

[B10] Brotto M. Bonewald L. (2015). Bone and muscle: interactions beyond mechanical. Bone 80, 109–114. doi: 10.1016/j.bone.2015.02.010 26453500 PMC4600532

[B37] Bu S. Chen Y. Wang S. Zhang F. Ji G. (2012). Treadmill training regulates β-catenin signaling through phosphorylation of GSK-3β in lumbar vertebrae of ovariectomized rats. Eur. J. Appl. Physiol. 112, 3295–3304. doi: 10.1007/s00421-011-2306-4 22252247

[B88] Buck H. V. Torre O. M. Leser J. M. Gould N. R. Ward C. W. Stains J. P. (2024). Nitric oxide contributes to rapid sclerostin protein loss following mechanical load. Biochem. Biophys. Res. Commun. 727, 150315. doi: 10.1016/j.bbrc.2024.150315 38950493 PMC12784413

[B45] Capato L. L. C. Porto J. M. Jerônimo J. M. Ribeiro B. Ferrioli E. de Paula F. J. A. . (2023). Contribution of hip abductor muscles on bone mineral density and functionality in older women. J. Clin. Densitometry. 26(1), 97–103. doi: 10.1016/j.jocd.2022.12.007 36543669

[B27] Çergel Y. Topuz O. Alkan H. Sarsan A. Akkoyunlu N. S. (2019). The effects of short-term back extensor strength training in postmenopausal osteoporotic women with vertebral fractures: comparison of supervised and home exercise program. Arch. Osteoporosis 14, 82. doi: 10.1007/s11657-019-0632-z 31352573

[B19] Chen Z. Shi T. Li W. Sun J. Yao Z. Liu W. (2023). Role of paraspinal muscle degeneration in the occurrence and recurrence of osteoporotic vertebral fracture: a meta-analysis. Front. Endocrinol. (Lausanne) 13, 1073013. doi: 10.3389/fendo.2022.1073013 36686478 PMC9845601

[B36] Chen Y. Wang S. Bu S. Wang Y. Duan Y. Yang S. (2011). Treadmill training prevents bone loss by inhibition of PPARγ expression but not promoting of Runx2 expression in ovariectomized rats. Eur. J. Appl. Physiol. 111, 1759–1767. doi: 10.1007/s00421-010-1820-0 21221986

[B116] Chi G. Qiu L. Ma J. Wu W. Zhang Y. (2023). The association of osteoprotegerin and RANKL with osteoporosis: a systematic review with meta-analysis. J. Orthop. Surg. Res. 18, 839. doi: 10.1186/s13018-023-04179-5 37932757 PMC10629047

[B16] Chiapparelli E. Okano I. Adl Amini D. Zhu J. Salzmann S. N. Tan E. T. . (2022). The association between lumbar paraspinal muscle functional cross-sectional area on MRI and regional volumetric bone mineral density measured by quantitative computed tomography. Osteoporos Int. 33, 2537–2545. doi: 10.1007/s00198-022-06430-x 35933479

[B76] Choi J. U. A. Kijas A. W. Lauko J. Rowan A. E. (2022). The mechanosensory role of osteocytes and implications for bone health and disease states. Front. Cell Dev. Biol. 9, 770143. doi: 10.3389/fcell.2021.770143 35265628 PMC8900535

[B107] Chowdhury S. Schulz L. Palmisano B. Singh P. Berger J. M. Yadav V. K. . (2020). Muscle-derived interleukin 6 increases exercise capacity by signaling in osteoblasts. J. Clin. Invest. 130, 2888–2902. doi: 10.1172/JCI133572 32078586 PMC7260002

[B1] Clynes M. A. Gregson C. L. Bruyère O. Cooper C. Dennison E. M. (2021). Osteosarcopenia: where osteoporosis and sarcopenia collide. Rheumatol. (Oxford) 60, 529–537. doi: 10.1093/rheumatology/keaa755 33276373

[B101] Colaianni G. Cuscito C. Mongelli T. Pignataro P. Buccoliero C. Liu P. . (2015). The myokine irisin increases cortical bone mass. Proc. Natl. Acad. Sci. U.S.A. 112, 12157–12162. doi: 10.1073/pnas.1516622112 26374841 PMC4593131

[B3] Compston J. E. McClung M. R. Leslie W. D. (2019). Osteoporosis. Lancet 393, 364–376. doi: 10.1016/S0140-6736(18)32112-3 30696576

[B4] Cruz-Jentoft A. J. Bahat G. Bauer J. Boirie Y. Bruyère O. Cederholm T. . (2019). Sarcopenia: revised European consensus on definition and diagnosis. Age Ageing 48, 16–31. doi: 10.1093/ageing/afy169 30312372 PMC6322506

[B99] Drey M. Sieber C. C. Bertsch T. Bauer J. M. Schmidmaier R. (2016). Osteosarcopenia is more than sarcopenia and osteopenia alone. Aging Clin. Exp. Res. 28, 895–899. doi: 10.1007/s40520-015-0494-1 26563287

[B6] Frost H. M. (2003). Bone's mechanostat: a 2003 update. Anat. Rec. A. Discov. Mol. Cell. Evol. Biol. 275, 1081–1101. doi: 10.1002/ar.a.10119 14613308

[B59] Fujii N. Imura T. Mitsutake T. Hirohama K. Okimoto N. Tsukamoto M. . (2026). Effects of exercise on the cortical bone of the proximal femur in women: a systematic review and meta-analysis. Bone 203, 117740. doi: 10.1016/j.bone.2025.117740 41317827

[B39] Gala J. Díaz-Curiel M. de la Piedra C. Calero J. (2001). Short- and long-term effects of calcium and exercise on bone mineral density in ovariectomized rats. Br. J. Nutr. 86, 521–527. doi: 10.1079/bjn2001428 11591240

[B38] Gao L. Li Y. Yang Y. J. Zhang D. Y. (2021). The effect of moderate-intensity treadmill exercise on bone mass and the transcription of peripheral blood mononuclear cells in ovariectomized rats. Front. Physiol. 12, 729910. doi: 10.3389/fphys.2021.729910 34777002 PMC8589120

[B52] Ge Y. Chen Y. Liu G. Zhu S. Li B. Tian M. . (2023). Association between hip bone mineral density and mortality risk after hip fracture: a prospective cohort study. Calcif. Tissue Int. 113, 295–303. doi: 10.1007/s00223-023-01109-9 37347299 PMC10449952

[B5] Goodpaster B. H. Park S. W. Harris T. B. Kritchevsky S. B. Nevitt M. Schwartz A. V. . (2006). The loss of skeletal muscle strength, mass, and quality in older adults: the Health, Aging and Body Composition Study. J. Gerontol A. Biol. Sci. Med. Sci. 61, 1059–1064. doi: 10.1093/gerona/61.10.1059 17077199

[B87] Gould N. R. Williams K. M. Joca H. C. Torre O. M. Lyons J. S. Leser J. M. . (2021). Disparate bone anabolic cues activate bone formation by regulating the rapid lysosomal degradation of sclerostin protein. eLife 10, e64393. doi: 10.7554/eLife.64393 33779549 PMC8032393

[B17] Han G. Zou D. Liu Z. Zhou S. Li W. Gong C. . (2022). Paraspinal muscle characteristics on MRI in degenerative lumbar spine with normal bone density, osteopenia and osteoporosis: a case-control study. BMC Musculoskelet. Disord. 23, 73. doi: 10.1186/s12891-022-05036-y 35057764 PMC8780389

[B110] Hang K. Ye C. Xu J. Chen E. Wang C. Zhang W. . (2019). Apelin enhances the osteogenic differentiation of human bone marrow mesenchymal stem cells partly through Wnt/β-catenin signaling pathway. Stem Cell Res. Ther. 10, 189. doi: 10.1186/s13287-019-1286-x 31238979 PMC6593611

[B61] Hartley C. Folland J. P. Kerslake R. Brooke-Wavell K. (2020). High-impact exercise increased femoral neck bone density with no adverse effects on imaging markers of knee osteoarthritis in postmenopausal women. J. Bone Miner. Res. 35, 53–63. doi: 10.1002/jbmr.3867 31498922

[B57] Hejazi K. Askari R. Hofmeister M. (2022). Effects of physical exercise on bone mineral density in older postmenopausal women: a systematic review and meta-analysis of randomized controlled trials. Arch. Osteoporos 17, 102. doi: 10.1007/s11657-022-01140-7 35896850

[B98] Hirschfeld H. P. Kinsella R. Duque G. (2017). Osteosarcopenia: where bone, muscle, and fat collide. Osteoporos Int. 28, 2781–2790. doi: 10.1007/s00198-017-4151-8 28733716

[B26] Hongo M. Itoi E. Sinaki M. Miyakoshi N. Shimada Y. Maekawa S. . (2007). Effect of low-intensity back exercise on quality of life and back extensor strength in patients with osteoporosis: a randomized controlled trial. Osteoporosis Int. 18, 1389–1395. doi: 10.1007/s00198-007-0398-9 17572835

[B79] Hu Y. J. Wu X. Wang F. Jin Y. Jin Y. Liu Y. . (2025). Piezo1-mediated mechanotransduction controls osteocyte maturation and dendrite development via a YAP-CCN-Src signaling axis. Nat. Commun. 16, 10859. doi: 10.1038/s41467-025-65636-9 41315189 PMC12675637

[B47] Huang P. Ge Y. Liu Y. Geng J. Zhang W. Liang W. . (2024). Association between trochanteric fractures and gluteal muscle size, density in older women: a cross-sectional study at a university hospital. BMJ Open 14, e086855. doi: 10.1136/bmjopen-2024-086855 39645269 PMC11535699

[B103] Jedrychowski M. P. Wrann C. D. Paulo J. A. Gerber K. K. Szpyt J. Robinson M. M. . (2015). Detection and quantitation of circulating human irisin by tandem mass spectrometry. Cell Metab. 22, 734–740. doi: 10.1016/j.cmet.2015.08.001 26278051 PMC4802359

[B96] Karsenty G. Mera P. (2018). Molecular bases of the crosstalk between bone and muscle. Bone 115, 43–49. doi: 10.1016/j.bone.2017.04.006 28428077 PMC6557139

[B56] Kelley G. A. Kelley K. S. (2006). Exercise and bone mineral density at the femoral neck in postmenopausal women: a meta-analysis of controlled clinical trials with individual patient data. Am. J. Obstet. Gynecol 194, 760–767. doi: 10.1016/j.ajog.2005.09.006 16522410

[B29] Kemmler W. Kohl M. Fröhlich M. Jakob F. Engelke K. von Stengel S. . (2020). Effects of high-intensity resistance training on osteopenia and sarcopenia parameters in older men with osteosarcopenia: one-year results of the randomized controlled Franconian Osteopenia and Sarcopenia Trial (FrOST). J. Bone Miner. Res. 35, 1634–1644. doi: 10.1002/jbmr.4027 32270891

[B102] Kim H. Wrann C. D. Jedrychowski M. P. Vidoni S. Kitase Y. Nagano K. . (2018). Irisin mediates effects on bone and fat via αV integrin receptors. Cell. 175, 1756–1768.e17. doi: 10.1016/j.cell.2018.10.025 30550785 PMC6298040

[B20] Kim H. J. Yang J. H. Chang D. G. Suk S. I. Suh S. W. Song K. S. . (2023). Significance of paraspinal muscle quality in risk between single and multiple osteoporotic vertebral fractures. Eur. Spine J. 32, 1763–1770. doi: 10.1007/s00586-023-07670-z 36977941

[B30] Kircher K. Chaudry O. Nagel A. M. Ghasemikaram M. Uder M. Jakob F. . (2024). Effects of high-intensity training on fatty infiltration in paraspinal muscles in elderly males with osteosarcopenia – the randomized controlled FrOST study. BMC Geriatrics 24, 141. doi: 10.1186/s12877-024-04736-5 38326734 PMC10851592

[B97] Kirk B. Feehan J. Lombardi G. Duque G. (2020). Muscle, bone, and fat crosstalk: the biological role of myokines, osteokines, and adipokines. Curr. Osteoporos Rep. 18, 388–400. doi: 10.1007/s11914-020-00599-y 32529456

[B60] Kistler-Fischbacher M. Yong J. S. Weeks B. K. Beck B. R. (2022). High-intensity exercise and geometric indices of hip bone strength in postmenopausal women on or off bone medication: the MEDEX-OP randomised controlled trial. Calcif. Tissue Int. 111, 256–266. doi: 10.1007/s00223-022-00991-z 35690931 PMC9188729

[B108] Kitase Y. Vallejo J. A. Gutheil W. Vemula H. Jahn K. Yi J. . (2018). β-Aminoisobutyric acid, L-BAIBA, is a muscle-derived osteocyte survival factor. Cell Rep. 22, 1531–1544. doi: 10.1016/j.celrep.2018.01.041 29425508 PMC5832359

[B89] Lawson L. Y. Migotsky N. Chermside-Scabbo C. J. Shuster J. T. Joeng K. S. Civitelli R. . (2022). Loading-induced bone formation is mediated by Wnt1 induction in osteoblast-lineage cells. FASEB J. 36, e22502. doi: 10.1096/fj.202200591R 35969160 PMC9430819

[B94] Leuchtmann A. B. Adak V. Dilbaz S. Handschin C. (2021). The role of the skeletal muscle secretome in mediating endurance and resistance training adaptations. Front. Physiol. 12, 709807. doi: 10.3389/fphys.2021.709807 34456749 PMC8387622

[B69] Lewis K. J. Frikha-Benayed D. Louie J. Stephen S. Spray D. C. Thi M. M. . (2017). Osteocyte calcium signals encode strain magnitude and loading frequency *in vivo*. Proc. Natl. Acad. Sci. U.S.A. 114, 11775–11780. doi: 10.1073/pnas.1707863114 29078317 PMC5676898

[B18] Li Z. Chen J. Yang J. Wang R. Wang W. (2024). Relationship between paraspinal muscle properties and bone mineral density based on QCT in patients with lumbar disc herniation. BMC Musculoskelet. Disord. 25, 360. doi: 10.1186/s12891-024-07484-0 38714980 PMC11075372

[B74] Li J. Wang Y. Zhang X. Zhang P. Su Y. Bai L. . (2022). Associations of muscle size and fatty infiltration with bone mineral density of the proximal femur bone. Front. Endocrinol. (Lausanne) 13, 990487. doi: 10.3389/fendo.2022.990487 36237187 PMC9552015

[B82] Li X. Zhang Y. Kang H. Liu W. Liu P. Zhang J. . (2005). Sclerostin binds to LRP5/6 and antagonizes canonical Wnt signaling. J. Biol. Chem. 280, 19883–19887. doi: 10.1074/jbc.M413274200 15778503

[B119] Li G. Zhang L. Ning K. Yang B. Acosta F. M. Shang P. . (2021). Osteocytic connexin43 channels regulate bone-muscle crosstalk. Cells 10, 237. doi: 10.3390/cells10020237 33530465 PMC7911162

[B95] Li G. Zhang L. Wang D. AIQudsy L. Jiang J. X. Xu H. . (2019). Muscle-bone crosstalk and potential therapies for sarco-osteoporosis. J. Cell. Biochem. 120, 14262–14273. doi: 10.1002/jcb.28946 31106446 PMC7331460

[B14] Li X. Zhang Y. Xie Y. Lu R. Tao H. Chen S. (2022). Correlation between bone mineral density (BMD) and paraspinal muscle fat infiltration based on QCT: a cross-sectional study. Calcif. Tissue Int. 110, 666–673. doi: 10.1007/s00223-022-00944-6 35006307

[B54] Libouban H. Guintard C. Minier N. Aguado E. Chappard D. (2018). Long-term quantitative evaluation of muscle and bone wasting induced by botulinum toxin in mice using microcomputed tomography. Calcif. Tissue Int. 102, 695–704. doi: 10.1007/s13105-021-00838-5 29222689

[B111] Liu M. Han Y. Wang J. Zhu Y. Zhang Y. Chu Q. . (2025). Skeletal muscle-derived IL-33 mediates muscle-to-bone crosstalk and regulates bone metabolism via CD8+ T cell-secreted CCL5. EBioMedicine 122, 106024. doi: 10.1016/j.ebiom.2025.106024 41237669 PMC12661402

[B73] Lu Y. Xu Z. Wang L. Li W. Zhao Y. Su Y. . (2020). Associations of muscle size and density with proximal femur bone in a community dwelling older population. Front. Endocrinol. (Lausanne) 11, 503. doi: 10.3389/fendo.2020.00503 32849289 PMC7399084

[B85] Lyons J. S. Joca H. C. Law R. A. Williams K. M. Kerr J. P. Shi G. . (2017). Microtubules tune mechanotransduction through NOX2 and TRPV4 to decrease sclerostin abundance in osteocytes. Sci. Signal. 10, eaan5748. doi: 10.1126/scisignal.aan5748 29162742 PMC5858867

[B12] Ma H. T. Griffith J. F. Xu L. Leung P. C. (2014). The functional muscle-bone unit in subjects of varying BMD. Osteoporos Int. 25, 999–1004. doi: 10.1007/s00198-013-2482-7 24030288

[B112] Ma S. Xing X. Huang H. Gao X. Lu Y. Yang J. . (2023). Skeletal muscle-derived extracellular vesicles transport glycolytic enzymes to mediate muscle-to-bone crosstalk. Cell Metab. 35, 2028–2043.e7. doi: 10.1016/j.cmet.2023.10.013 37939660

[B48] Ma H. Zhong Y. Ye X. Peng Z. Lin M. Xu X. . (2026). Association of computed tomography-based radiodensity of the peri-hip muscles with hip fracture type and severity: a retrospective study in middle-aged and elderly patients. BMC Musculoskelet. Disord. 27, 380. doi: 10.1186/s12891-026-09764-3 41888832 PMC13141344

[B75] Machireddy M. Oberman A. G. DeBiase L. Stephens M. Li J. Littlepage L. E. . (2024). Controlled mechanical loading affects the osteocyte transcriptome in porcine trabecular bone in situ. Bone 181, 117028. doi: 10.1016/j.bone.2024.117028 38309412 PMC10923013

[B23] Mai C. Liu Y. Xu D. Geng J. Wang W. Zhu K. . (2024). Role of effective atomic number of paraspinal muscles in the prediction of acute vertebral fracture risk assessment: a cross-sectional case-control study. Br. J. Radiol. 97, 1437–1442. doi: 10.1093/bjr/tqae112 38833675 PMC11256961

[B113] Mera P. Laue K. Ferron M. Confavreux C. Wei J. Galán-Díez M. . (2016a). Osteocalcin signaling in myofibers is necessary and sufficient for optimum adaptation to exercise. Cell Metab. 23, 1078–1092. doi: 10.1016/j.cmet.2016.05.004 27304508 PMC4910629

[B114] Mera P. Laue K. Wei J. Berger J. M. Karsenty G. (2016b). Osteocalcin is necessary and sufficient to maintain muscle mass in older mice. Mol. Metab. 5, 1042–1047. doi: 10.1016/j.molmet.2016.07.002 27689017 PMC5034485

[B31] Mohebbi R. Shojaa M. Kohl M. von Stengel S. Jakob F. Kerschan-Schindl K. . (2023). Exercise training and bone mineral density in postmenopausal women: an updated systematic review and meta-analysis of intervention studies with emphasis on potential moderators. Osteoporos Int. 34, 1145–1178. doi: 10.1007/s00198-023-06682-1 36749350 PMC10282053

[B58] O’Bryan S. J. Giuliano C. Woessner M. N. Vogrin S. Smith C. Duque G. . (2022). Progressive resistance training for concomitant increases in muscle strength and bone mineral density in older adults: a systematic review and meta-analysis. Sports Med. 52, 1939–1960. doi: 10.1007/s40279-022-01675-2 35608815 PMC9325860

[B64] O’Rourke D. Beck B. R. Harding A. T. Watson S. L. Pivonka P. Martelli S. . (2023). Geometry and bone mineral density determinants of femoral neck strength changes following exercise. Biomech. Model. Mechanobiol. 22, 207–216. doi: 10.1007/s10237-022-01642-w 36271264 PMC9958140

[B44] Pasco J. A. Holloway K. L. Brennan-Olsen S. L. Moloney D. J. Kotowicz M. A. (2015). Muscle strength and areal bone mineral density at the hip in women: a cross-sectional study. BMC Musculoskeletal Disord. 16, 124. doi: 10.1186/s12891-015-0586-2 26003407 PMC4493811

[B92] Pedersen B. K. Febbraio M. A. (2012). Muscles, exercise and obesity: skeletal muscle as a secretory organ. Nat. Rev. Endocrinol. 8, 457–465. doi: 10.1038/nrendo.2012.49 22473333

[B53] Peng Z. Q. Tuukkanen J. Väänänen H. K. (1994). Exercise can provide protection against bone loss and prevent the decrease in mechanical strength of femoral neck in ovariectomized rats. J. Bone Miner. Res. 9, 1559–1564. doi: 10.1002/jbmr.5650091008 7817801

[B33] Poliachik S. L. Khokhlova T. D. Wang Y. N. Simon J. C. Bailey M. R. (2014). Pulsed focused ultrasound treatment of muscle mitigates paralysis-induced bone loss in the adjacent bone: a study in a mouse model. Ultrasound Med. Biol. 40, 2113–2124. doi: 10.1016/j.ultrasmedbio.2014.02.027 24857416 PMC4410740

[B81] Poole K. E. S. van Bezooijen R. L. Loveridge N. Hamersma H. Papapoulos S. E. Löwik C. W. G. M. . (2005). Sclerostin is a delayed secreted product of osteocytes that inhibits bone formation. FASEB J. 19, 1842–1844. doi: 10.1096/fj.05-4221fje 16123173

[B65] Prowse J. Jaiswal S. Gentle J. Sorial A. K. Witham M. D. (2024). Feasibility, acceptability and prognostic value of muscle mass and strength measurement in patients with hip fracture: a systematic review. Eur. Geriatr. Med. 15, 1603–1614. doi: 10.1007/s41999-024-01102-x 39614068 PMC11632060

[B100] Qin Y. Peng Y. Zhao W. Pan J. Ksiezak-Reding H. Cardozo C. . (2017). Myostatin inhibits osteoblastic differentiation by suppressing osteocyte-derived exosomal microRNA-218: a novel mechanism in muscle-bone communication. J. Biol. Chem. 292, 11021–11033. doi: 10.1074/jbc.M116.770941 28465350 PMC5491785

[B11] Reginster J. Y. Beaudart C. Buckinx F. Bruyère O. (2016). Osteoporosis and sarcopenia: two diseases or one? Curr. Opin. Clin. Nutr. Metab. Care 19, 31–36. doi: 10.1097/MCO.0000000000000230 26418824 PMC4888925

[B24] Revel M. Mayoux-Benhamou M. A. Rabourdin J. P. Bagheri F. Roux C. (1993). One-year psoas training can prevent lumbar bone loss in postmenopausal women: a randomized controlled trial. Calcif. Tissue Int. 53, 307–311. doi: 10.1007/BF01351834 8287317

[B67] Robling A. G. Hinant F. M. Burr D. B. Turner C. H. (2002). Improved bone structure and strength after long-term mechanical loading is greatest if loading is separated into short bouts. J. Bone Miner. Res. 17, 1545–1554. doi: 10.1359/jbmr.2002.17.8.1545 12162508

[B83] Robling A. G. Niziolek P. J. Baldridge L. A. Condon K. W. Allen M. R. Alam I. . (2008). Mechanical stimulation of bone *in vivo* reduces osteocyte expression of Sost/sclerostin. J. Biol. Chem. 283, 5866–5875. doi: 10.1074/jbc.M705092200 18089564

[B41] Sato C. Miyakoshi N. Kasukawa Y. Nozaka K. Tsuchie H. Nagahata I. . (2021). Teriparatide and exercise improve bone, skeletal muscle, and fat parameters in ovariectomized and tail-suspended rats. J. Bone Miner. Metab. 39, 385–395. doi: 10.1007/s00774-020-01184-0 33392725

[B8] Schoenau E. Neu C. M. Beck B. Manz F. Rauch F. (2002). Bone mineral content per muscle cross-sectional area as an index of the functional muscle-bone unit. J. Bone Miner. Res. 17, 1095–1101. doi: 10.1359/jbmr.2002.17.6.1095 12054165

[B7] Schönau E. (2005). From mechanostat theory to development of the "Functional Muscle-Bone-Unit. J. Musculoskelet. Neuronal Interact. 5, 232–238. 16172514

[B42] Sehmisch S. Galal R. Kolios L. Tezval M. Dullin C. Zimmer S. . (2009). Effects of low-magnitude, high-frequency mechanical stimulation in the rat osteopenia model. Osteoporos Int. 20, 1999–2008. doi: 10.1007/s00198-009-0892-3 19283328 PMC2777215

[B72] Sen B. Xie Z. Case N. Ma M. Rubin C. Rubin J. (2008). Mechanical strain inhibits adipogenesis in mesenchymal stem cells by stimulating a durable β-catenin signal. Endocrinology 149, 6065–6075. doi: 10.1210/en.2008-0687 18687779 PMC2613068

[B93] Severinsen M. C. K. Pedersen B. K. (2020). Muscle-organ crosstalk: the emerging roles of myokines. Endocr. Rev. 41, 594–609. doi: 10.1210/endrev/bnaa016 32393961 PMC7288608

[B35] Simões P. A. Zamarioli A. Blóes P. Mazzocato F. C. Pereira L. H. A. Volpon J. B. . (2008). Effect of treadmill exercise on lumbar vertebrae in ovariectomized rats: anthropometrical and mechanical analyses. Acta Bioeng. Biomech. 10, 39–41. 19031996

[B25] Sinaki M. Itoi E. Wahner H. W. Wollan P. Gelzcer R. Mullan B. P. . (2002). Stronger back muscles reduce the incidence of vertebral fractures: a prospective 10-year follow-up of postmenopausal women. Bone 30, 836–841. doi: 10.1016/S8756-3282(02)00739-1 12052450

[B117] Sirikul W. Siri-Angkul N. Chattipakorn N. Chattipakorn S. C. (2022). Fibroblast growth factor 23 and osteoporosis: evidence from bench to bedside. Int. J. Mol. Sci. 23, 2500. doi: 10.3390/ijms23052500 35269640 PMC8909928

[B68] Srinivasan S. Weimer D. A. Agans S. C. Bain S. D. Gross T. S. (2002). Low-magnitude mechanical loading becomes osteogenic when rest is inserted between each load cycle. J. Bone Miner. Res. 17, 1613–1620. doi: 10.1359/jbmr.2002.17.9.1613 12211431 PMC1435731

[B22] Tang M. Zhang G. Zeng F. Chang X. Fang Q. He M. . (2024). Paraspinal muscle parameters' predictive value for new vertebral compression fractures post-vertebral augmentation: Nomogram development and validation. Front. Med. (Lausanne) 11, 1379078. doi: 10.3389/fmed.2024.1379078 38813387 PMC11133621

[B55] Tang L. Zhao T. Kang Y. An S. Fan X. Sun L. (2022). MSTN is an important myokine for weight-bearing training to attenuate bone loss in ovariectomized rats. J. Physiol. Biochem. 78, 61–72. doi: 10.1007/s13105-021-00838-5 34453705

[B2] Teng Z. Zhu Y. Teng Y. Long Q. Hao Q. Yu X. . (2021). The analysis of osteosarcopenia as a risk factor for fractures, mortality, and falls. Osteoporos Int. 32, 2173–2183. doi: 10.1007/s00198-021-05963-x 33877382

[B70] Thompson W. R. Rubin C. T. Rubin J. (2012). Mechanical regulation of signaling pathways in bone. Gene 503, 179–193. doi: 10.1016/j.gene.2012.04.076 22575727 PMC3371109

[B84] Tu X. Rhee Y. Condon K. W. Bivi N. Allen M. R. Dwyer D. . (2012). Sost downregulation and local Wnt signaling are required for the osteogenic response to mechanical loading. Bone 50, 209–217. doi: 10.1016/j.bone.2011.10.025 22075208 PMC3246572

[B66] Turner C. H. Robling A. G. (2003). Designing exercise regimens to increase bone strength. Exerc Sport Sci. Rev. 31, 45–50. doi: 10.1097/00003677-200301000-00009 12562170

[B71] Uda Y. Azab E. Sun N. Shi C. Pajevic P. D. (2017). Osteocyte mechanobiology. Curr. Osteoporos Rep. 15, 318–325. doi: 10.1007/s11914-017-0373-0 28612339 PMC5656287

[B109] Vinel C. Lukjanenko L. Batut A. Deleruyelle S. Pradère J. P. Le Gonidec S. . (2018). The exerkine apelin reverses age-associated sarcopenia. Nat. Med. 24, 1360–1371. doi: 10.1038/s41591-018-0131-6 30061698

[B115] Vitale J. A. Sansoni V. Faraldi M. Messina C. Verdelli C. Lombardi G. . (2021). Circulating carboxylated osteocalcin correlates with skeletal muscle mass and risk of fall in postmenopausal osteoporotic women. Front. Endocrinol. (Lausanne) 12, 669704. doi: 10.3389/fendo.2021.669704 34025583 PMC8133362

[B91] Wang B. Liu J. Wang Q. Arhatte M. Cheong L. Y. Glogowska E. . (2025). Piezo1 activation suppresses bone marrow adipogenesis to prevent osteoporosis by inhibiting a mechanoinflammatory autocrine loop. Signal. Transduct Target Ther. 10, 357. doi: 10.1038/s41392-025-02455-w 41145414 PMC12559722

[B34] Wang X. Wang S. Yan P. Bian Z. Li M. Hou C. . (2018). Paravertebral injection of botulinum toxin-A reduces lumbar vertebral bone quality. J. Orthop. Res. 36, 2664–2670. doi: 10.1002/jor.24029 29687610

[B51] Wang L. Yang M. Ge Y. Liu Y. Su Y. Guo Z. . (2023). Muscle size and density are independently associated with death after hip fracture: a prospective cohort study. J. Cachexia Sarcopenia Muscle. 14(4), 1824–1835. doi: 10.1002/jcsm.13261 37208980 PMC10401534

[B49] Wang L. Yang M. Ge Y. Liu Y. Wang G. Su Y. . (2022). Muscle density is an independent risk factor of second hip fracture: a prospective cohort study. J. Cachexia Sarcopenia Muscle. 13(3), 1927–1937. doi: 10.1002/jcsm.12996 35429146 PMC9178374

[B50] Wang L. Yang M. Ge Y. Liu Y. Wang G. Su Y. . (2024). Risk prediction of second hip fracture by bone and muscle density of the hip varies with time after first hip fracture: a prospective cohort study. Bone Rep. 20, 101732. doi: 10.1016/j.bonr.2023.101732 38226335 PMC10788229

[B46] Wang L. Yin L. Zhao Y. Su Y. Sun W. Liu Y. . (2020a). Muscle density discriminates hip fracture better than computed tomography X-ray absorptiometry hip areal bone mineral density. J. Cachexia Sarcopenia Muscle 11, 1799–1812. doi: 10.1002/jcsm.12616 32894000 PMC7749550

[B78] Wang L. You X. Lotinun S. Zhang L. Wu N. Zou W. (2020b). Mechanical sensing protein PIEZO1 regulates bone homeostasis via osteoblast-osteoclast crosstalk. Nat. Commun. 11, 282. doi: 10.1038/s41467-019-14146-6 31941964 PMC6962448

[B120] Waning D. L. Mohammad K. S. Reiken S. Xie W. Andersson D. C. John S. . (2015). Excess TGF-β mediates muscle weakness associated with bone metastases in mice. Nat. Med. 21, 1262–1271. doi: 10.1038/nm.3961 26457758 PMC4636436

[B28] Watson S. L. Weeks B. K. Weis L. J. Harding A. T. Horan S. A. Beck B. R. (2018). High-intensity resistance and impact training improves bone mineral density and physical function in postmenopausal women with osteopenia and osteoporosis: the LIFTMOR randomized controlled trial. J. Bone Miner. Res. 33, 211–220. doi: 10.1002/jbmr.3284 28975661

[B86] Williams K. M. Leser J. M. Gould N. R. Joca H. C. Lyons J. S. Khairallah R. J. . (2020). TRPV4 calcium influx controls sclerostin protein loss independent of purinergic calcium oscillations. Bone 136, 115356. doi: 10.1016/j.bone.2020.115356 32272228 PMC7605285

[B40] Wu Q. Zhong P. Ning P. Tan L. Huang X. Peng T. . (2022). Treadmill training mitigates bone deterioration via inhibiting NLRP3/Caspase1/IL-1β signaling in aged rats. BMC Musculoskelet. Disord. 23, 1089. doi: 10.1186/s12891-022-06055-5 36514079 PMC9746211

[B105] Xian L. Wu X. Pang L. Lou M. Rosen C. J. Qiu T. . (2012). Matrix IGF-1 maintains bone mass by activation of mTOR in mesenchymal stem cells. Nat. Med. 18, 1095–1101. doi: 10.1038/nm.2793 22729283 PMC3438316

[B21] Xiong Y. Zhang C. Chen X. Wu L. Liang S. Zhang Y. . (2024). Prediction of subsequent vertebral fracture after acute osteoporotic fractures from clinical and paraspinal muscle features. Calcif. Tissue Int. 114, 614–624. doi: 10.1007/s00223-024-01209-0 38714533 PMC11090933

[B77] Zarka M. Etienne F. Bourmaud M. Szondi D. Schwartz J. M. Kampmann K. . (2021). Mechanical loading activates the YAP/TAZ pathway and chemokine expression in the MLO-Y4 osteocyte-like cell line. Lab. Invest. 101, 1597–1604. doi: 10.1038/s41374-021-00668-5 34521992

[B13] Zhao Y. Huang M. Serrano Sosa M. Cattell R. Fan W. Li M. . (2019). Fatty infiltration of paraspinal muscles is associated with bone mineral density of the lumbar spine. Arch. Osteoporos 14, 99. doi: 10.1007/s11657-019-0639-5 31617017

[B118] Zhao D. Riquelme M. A. Guda T. Tu C. Xu H. Gu S. . (2022). Connexin hemichannels with prostaglandin release in anabolic function of bone to mechanical loading. eLife 11, e74365. doi: 10.7554/eLife.74365 35132953 PMC8824479

[B15] Zhou S. Chen S. Zhu X. You T. Li P. Shen H. . (2022). Associations between paraspinal muscles fatty infiltration and lumbar vertebral bone mineral density - An investigation by fast kVp switching dual-energy CT and QCT. Eur. J. Radiol. Open 9, 100447. doi: 10.1016/j.ejro.2022.100447 36277658 PMC9579482

[B80] Zhou T. Gao B. Fan Y. Liu Y. Feng S. Cong Q. . (2020). Piezo1/2 mediate mechanotransduction essential for bone formation through concerted activation of NFAT-YAP1-β-catenin. eLife 9, e52779. doi: 10.7554/eLife.52779 32186512 PMC7112954

